# Behavioral Changes After the COVID-19 Lockdown in Italy

**DOI:** 10.3389/fpsyg.2021.617315

**Published:** 2021-03-10

**Authors:** Veronica Cucchiarini, Laura Caravona, Laura Macchi, Federico L. Perlino, Riccardo Viale

**Affiliations:** ^1^Department of Psychology, University of Milano-Bicocca, Milan, Italy; ^2^Department of Sociology and Social Research, University of Milano-Bicocca, Milan, Italy; ^3^Department of Economics, Management and Statistics, University of Milano-Bicocca, Milan, Italy

**Keywords:** COVID-19, behavioral changes, social norms, message contents, risk perception, prevention behaviors

## Abstract

This study aims at identifying the tools necessary for COVID-19 health emergency management, with particular reference to the period following the first lockdown, a crucial phase in which it was important to favor the maintenance of protective behaviors. It also aims at identifying the messages and sources that were most effective in managing communication correctly in such a crucial phase that is likely characterized by a fall in perceived health risk (due to the flattening of the epidemic curve) and a simultaneous rise in perceived economic and social risks (due to the enduring calamity). Knowing what source will be most effective to convey a specific message is fundamental in enabling individuals to focus on and comply with the rules. At the same time, it is necessary to understand how the message should be presented, and the relationships between messages, sources and targets. To meet these goals, data were collected through a self-administered online questionnaire submitted to a sample of undergraduate students from a University in Lombardy–the region most affected by the pandemic in the first wave- (Study 1), and to a national sample composed of Italian citizens (Study 2). Through our first manipulation which explored the effectiveness of social norms in relation to different sources, we found that, in the national sample, the injunctive norm conveyed by the government was the most effective in promoting behavioral intentions. By contrast, among the students, results showed that for the critical group with a lower risk perception (less inclined to adopt prevention behavior) descriptive norms, which implicitly convey the risk perception of peers, were as effective as the government injunctive norm. Our second manipulation, identical in Study 1 and 2, compared four types of communication (emotional, exponential growth, both of them, or neutral). The neutral condition was the most memorable, but no condition was more effective than the others. Across all message types there was a high intention to adopt protective behaviors. The results indicate possible applicative implications of the adopted communicative tools.

## Introduction

In everyday life, it is rare to deal with numerically determined risks, and to tackle the uncertainties we face we mainly rely on our own experiences and data extracted from our environment, even if doing so produce severe distortions in judgment and decision making. The complexity of natural and social phenomena led Savage (1954) to distinguish between a small world and a large world. The former is characterized by the possibility of identifying relevant alternatives, consequences, and probabilities to explain and predict phenomena, while the latter, does not allow for this because a relevant part of information remains unknown. According to Savage, the large world is the realm of uncertainty, which can be of two different types depending on the phenomenon examined. Epistemic uncertainty “occurs when, ideally, empirical research and the collection of data are able to supply statistical figures that characterize relevant variables, their consequences, and probabilities” (Viale, 2020b). Ontic uncertainty (Gigerenzer and Gaissmaier, 2011; Couso and Dubois, 2014; Njå et al., 2017; Veraart et al., 2018), on the other hand, is required when empirical research is unable to determine the probability of an event occurring due to its complexity. In the case of a pandemic phenomenon such as COVID-19, uncertainty is epistemic, as research may be able to analyze and treat the evolution of the pandemic. In a situation of epistemic uncertainty, the treatment of the phenomenon depends, greatly, on the decisions taken in a heuristic and adaptive way (Gigerenzer et al., 1999), and on the progress of data collection, which, once the statistical risks have been identified, allows decision-makers to devise more appropriate measures to manage the emergency.

Another aspect to consider is that people have no previous experience as a reference. Additionally, the lack of reliable information about the nature, functioning, and ways of combating the infection has created a new situation, in which data from our environment are unreliable. The management of COVID-19 by the main world leaders has been varied, and subsequently, so have been the results of the more or less rapid implementation of containment measures. However, the time gap between the phases of COVID-19 infection management in different countries around the world has made it possible to use the knowledge previously acquired by others to develop strategies to promote desired behavioral changes. The mitigation actions that governments have to adopt in the containment of the COVID-19 pandemic must deal with the public risk perception, which affects people’s lifestyles, habits, and feelings. Wise et al. (2020) demonstrated that there was a sudden increase in risk perception during the initial phase of the COVID-19 pandemic because of the public health messages disseminated by the United States Government and media, which also proved effective in decreasing the tendency to be optimistic. The authors emphasized the importance of clear risk communication (for example, target-specific interventions to promote education on the beneficial effects of protective behaviors) to develop an accurate risk perception and, therefore, a more significant commitment to protective behaviors.

Risky situations are almost always accompanied by emotional reactions, which inevitably play a role in risk perceptions (e.g., risk as feelings: Loewenstein et al., 2001; Affect Heuristic: Slovic et al., 2004). Emotional reactions act as powerful motivators of behavior, such as practicing social distancing, hand washing, and supporting harsh policies (Frijda, 1986). However, these emotional reactions often diverge from cognitive evaluations and lead people to ignore crucial numeric information, such as probabilities (Rottenstreich and Hsee, 2001). Consequently people tend to rely on their feelings as a substitute for other information, such as numeric risk.

In general, we estimate the probability of an event as more likely to be high-risk if it receives strong media attention and if it has a high emotional impact. The information communicated by the media tends to promote feelings of danger and risk, such as those related to the availability heuristic (Tversky and Kahneman, 1973). Even if positive and negative information is communicated–for example, during the growing phase of the contagion of COVID-19, the percentage of people who died, survived or only had mild symptoms–people tend to focus disproportionately on the negative information (Baumeister et al., 2001; Tierney and Baumeister, 2019). It would be more effective to present information focusing on specific evidence to render complex content understandable and usable by decision-makers (Peters, 2017). In a recent study, Motta Zanin et al. (2020) demonstrate that better awareness about the COVID-19 emergency led to a higher level of acceptance of the more stringent containment measures. Moreover, individuals who informed themselves mainly through newspapers have a higher degree of knowledge than those who used television and social media. Social media has also widely promoted incorrect information (Frenkel et al., 2020).

The Behavioral Research Unit, headed by Lunn et al. (2020), investigated the effectiveness of two different communication strategies to promote social distancing behavior by focusing on the emotional aspects or the explanation of the transmission rate of COVID-19. The first strategy highlights the possibility of infecting specific individuals who are especially vulnerable to COVID-19. According to previous research (i.e., Jenni and Loewenstein, 1997; Lee and Feeley, 2016), people are more likely to make sacrifices to help specifically identified individuals rather than statistically described individuals. Small and Loewenstein (2003) also found this effect when an individual remains anonymous because it could induce stronger caring emotions. The second communication strategy focuses on the exponential nature of network transmission, highlighting the possibility that individual behavior results in multiple onward infections. Individuals have difficulty in accurately perceiving exponential growth and are inclined to underestimate it (exponential growth bias; Wagenaar and Sagaria, 1975). Communication that highlights exponential growth can increase the likelihood that people will recognize it, overcome it and behave accordingly (Witte, 1992). Their results illustrate that both experimental conditions have a greater effect in promoting infection containment behaviors than the control condition, where respecting social distancing at different moments of daily life is simply communicated.

During the lockdown period in Italy and other countries, public decision-makers could not coercively oblige people to follow specific basic prescriptions, for example, frequently washing their hands. Restrictive measures imposed by law (wearing masks, social distancing, and leaving home only out of necessity) had to find support based on social, behavioral prescriptions that reinforced their prevention function. Hence, legislative restrictions would have easily been ignored if citizens did not have a clear social perception of the pandemic situation. Social norms were found to be particularly relevant concerning health behaviors, and their usefulness was recognized by scholars from the earliest moments of the pandemic (e.g., Betsch, 2020; Olivera-La Rosa et al., 2020; Van Bavel et al., 2020).

The use of social norms by public institutions can have different natures and objectives from a behavioral point of view. This could involve the correction of erroneous perceptions and intentions of certain behaviors mainly related to health care–e.g., handwashing (Dickie et al., 2018) or alcohol consumption (Moreira et al., 2009)–but also to social problems–e.g., gender inequalities (Alon et al., 2020). Alternatively, specific norm-nudges (Sunstein, 2014; Bicchieri and Dimant, 2019) could be used to push people to behave in a non-coercive way by following the precautionary measures adopted institutionally (Van Bavel et al., 2020).

Classical social psychology studies have demonstrated that the effectiveness of a social norm largely depends on the perceived specificity of the normative content and on the degree of attentive focus that the norm can generate (Cialdini et al., 1990, 1991; Goldstein et al., 2008). According to Cialdini et al. (1991), norms can influence behavior only when the subject focuses on them as salient at the level of attention processes. However, if the risk is perceived as terrifying, possible defense mechanisms that may be used are removal and minimization (Festinger, 1957; Cooper, 2007). Individuals rely on experience and data extracted from their small environment, which can result in serious distortions of judgment and decisions leading to a very underestimated perception of risk (e.g., Newell et al., 2016). If the disconnection between perceived risk and real risk is too high, in both directions, then the usual winning behavioral norms may lose effectiveness. This ambivalence toward terrifying phenomena could be explained by the different propensity to riskexplained by a series of individual differences (Viale, 2020a), which include, for example, personality profiles, biological age, expertise, the salience of emotional characteristics, and non-explicit cognitive characteristics.

Several factors that make the use of social norms complex have recently been investigated in relation to the pandemic. A survey conducted at the University of Bolzano (Briscese et al., 2020) sought to investigate the relationship between people’s willingness to isolate themselves and their expectations regarding the duration of restrictive measures. When expectations are positive, that is, when people estimate that the restrictive measures will last less than expected, the willingness to practice social distancing increases; conversely, it decreases. Another study (Bilancini et al., 2020) conducted during the pandemic seeks to analyze the impact of social norms (personal, descriptive, and injunctive) promoted by leaflets on people’s behavior. The feedback provided by this study illustrates that the desired nudge effect was not obtained. In order for the rules to be more effective, the authors argued that it is necessary to test nudges that are stronger from the point of view of the emotional impact they can generate, for example, interventions that make use of shocking images.

Social norms could affect behaviors, particularly in the crucial phase that followed the first lockdown, when restrictions were removed. To understand how and what to communicate during this critical phase, it is also necessary to understand the imaginary representation related to the infection in the present situation, and how this is evolving. A recent study (Barrios and Hochberg, 2020) demonstrated that, for example, Trump voters in the United States have a lower perception of risk and are less committed to practicing social distancing. Their behaviors persist until official federal guidelines enforce social distancing. Furthermore, Brzezinski et al. (2020) focus on attitudes toward science. Compliance with social distancing policies can be influenced by beliefs about science and scientific consensus arguments.

The effectiveness of different messages may depend on who communicates them to whom. Tacit knowledge, implicit presuppositions, and implications are the necessary background of any kind of communication, and their consideration influences the degree of efficacy of a discourse, communication, and behavioral intervention (Bagassi and Macchi, 2016; Macchi and Bagassi, 2019). As previously said, several factors can influence the effectiveness of social norms. Chung and Rimal (2016) conceive these factors as moderators. It is plausible to expect that, in this case, one of the main factors of moderation among those identified by the authors is media exposure, which could lead some prescriptions to be more or less effective. Therefore, understanding how to intervene at a behavioral level using social norms, in a framework of such complexity, becomes extremely relevant.

Ali et al. (2020) showed in their study that many beliefs and knowledge related to COVID-19 were significantly predicted by the source of information, which was determined by the participants’ socio-demographic characteristics. Fridman et al. (2020) also pointed out how important it is to consider different information sources to ensure that diverse populations can access critical knowledge about COVID-19. Their study suggested that trust in sources could vary in relation to age and gender. These findings and those deriving from many other studies (e.g., Mohamad et al., 2020; Chu et al., 2021) highlight the importance of investigating the role of different sources in communicating information.

In the light of what has emerged from the literature on the phenomenon so far, the present paper aims at identifying the social norms, sources and contents that would be most effective in promoting prevention behaviors in this crucial phase -between two waves- of the pandemic, in which the contagion declines, legislative norms are loosened, and there is a risk of relapse. In particular, our two studies have been carried out in Italy between the last week of the first lockdown and the beginning of the post-lockdown–the so-called phase 2. We chose to focus on this specific phase since it is the crucial phase in which decision-making lies in the hands of individuals. Study 1 focuses on a national representative sample composed of Italian citizens of legal age, while Study 2 involves a sample of undergraduate students from the University of Milano-Bicocca, situated in Lombardy which was the region most affect by the pandemic during that period. The first objective of our studies is to identify the social norms and sources that would be most effective in preventing contagion behaviors during phase 2 in order to enable individuals to focus on and comply with the rules. In order to achieve this goal, risk perception and trust in sources are considered in the model since, as stated above, these variables play a role in influencing the effectiveness of social norms. It is important to underline that our first manipulation differs in Study 1 and Study 2 principally in the sources investigated and in the specific behavior promoted. It is not our intention to directly compare this manipulation between the two studies but instead to find “for each targeted sample” the most effective way to encourage commitment to protective behaviors. The second objective of our research is to gather information on how the communicated message should be presented: focusing on neutral, emotional, or exponential growth (or both) aspects. In this case the stimuli presented are equal for the two groups of participants. As shown above, emotional aspects and numerical information can significantly influence the effects of communication. We wonder whether at a time when we are overloaded with often contradictory information emphasizing one of these specific aspects can influence the focus of attention in adopting preventive behaviors.

## Study 1

Study 1 was conducted on a representative sample of the Italian population. It aims at firstly investigating which is the best source and norm to promote a specific preventive behavior (manipulation 1), in particular “to minimize verbal exchanges in indoor public places.” This message was primarily chosen because at the beginning of the COVID-19 pandemic, in Italy, wearing protective masks was recommended but not compulsory; hence, minimizing verbal exchanges between people was a primary way of preventing contagion. The sources of information identified (the Government and Scientists) are those that, at that time, were most concerned with prescribing behavior to avoid infection. Our second manipulation attempted to assess whether messages referencing either emotions or exponential growth, or both combined, or a neutral message referencing neither of these two aspects are most effective in influencing precautionary behaviors, such as practicing social distancing, using personal protective equipment, and washing one’s hands frequently. To achieve this aim, we based our stimuli on a cartoon promoted by the Italian Ministry of Health, which urged citizens to adopt infection-preventing behaviors.

### Methods

#### Participants

Four-hundred Italian citizens (aged between 18 and 70 years) were recruited by a market research company (Doxa) using stratified sampling by gender, geographic area, and town size. The sample includes 18–70 year-old participants (mean age = 45.85; SD = 12.71) living in different regions of Italy, which were characterized by different levels of contagion (low, medium, and high) during the lockdown. The participants were remunerated for undertaking the 20-min online study, and their socio-demographic information is summarized in Table 1. A further 276 subjects (41%) were excluded from the analysis because either they claimed to have contracted COVID-19 or were in close contact with people who had contracted it.

**TABLE 1:** Socio-demographic information of the national sample.

		*N* (%)
Gender	Male	199 (49.8%)
	Female	201 (50.2%)
Geographic Area	North–West	105 (26.3%)
	North–East	77 (19.3%)
	Center	83 (20.8%)
	South and Islands	135 (33.8%)
Age	18–30	55 (13.8%)
	31–45	142 (35.5%)
	46–60	141 (35.3%)
	60–70	62 (15.5%)
Education	Below degree	266 (66.5%)
	Degree or above	134 (33.5%)
Contagion Area	Regions with low contagion	108 (27.0%)
	Regions with medium contagion	114 (28.5%)
	Regions with high contagion	178 (44.5%)

#### Materials and Design

The survey data were collected through a self-administered questionnaire accessible through an online platform. The survey was administered to the national sample, from April 27 to April 30, during the last week of total lockdown in Italy. The questionnaire was composed of different parts, that were structured as follows:

(1) Awareness of the behaviors relevant to COVID-19 infection prevention (*open-ended question: “please list in order of importance the behaviors that you think are relevant to prevent covid-19 infection”*);
(2) Mental representation of the infection and consequences of the virus (*(1) “When you think of COVID-19 infection, what is the first word that comes to mind?” (2) “Let us talk about the effects and consequences of COVID-19 in general. Read the following words and choose the ones you think are most likely to be associated with COVID-19 infection: flue, war, plague, government conspiracy, Chernobyl, biological weapon, holidays, spare time, natural cycle, occasion, solidarity, enclosure, spiritual retreat”*);
(3) Comparison between the lockdown, the after lockdown, and the return to ordinary life: essential and non-essential behaviors and behavioral intentions (*(1) “Referring to the last week of lockdown, please indicate the number of times you went out to* […],” *(2) “Referring instead to phase two, please indicate the number of times you think you will go out, on average in 1 week, to* […],” *(3) “Referring to the period when you think we will return to ordinary life, please indicate the number of times you think you will go out, on average in 1 week, to* […]”).
(4) Time estimate to return to ordinary life (*“When do you think we will return to ordinary life? i.e., when social distancing will not be imposed anymore. Please indicate the number of months”*).
(5) Health, economic, privacy, and mobility risk perception (see Appendix 2);
(6) Trust in communication sources and emergency management (*“Indicate how much you trust the following sources, in relation to the current situation”*).

Each of these parts included specific questions answered by participants on a Likert scale (ranging from 1 to 7).

#### Manipulation 1: Norms and Sources

The first manipulation aimed at identifying which norm is the most effective and which source is the most appropriate in promoting a specific behavioral intention, taking into account the confidence in the communication sources.

The behavior to promote was “to minimize verbal exchanges in indoor public places.” Two different types of norms (Cialdini et al., 1990, 1991; Goldstein et al., 2008) were taken into consideration: the *injunctive norm* (“*it is necessary to*”) and the *descriptive local norm* (“*the inhabitants of your neighborhood [*…*]”*). The injunctive norms refer to individuals’ perceptions of what is socially acceptable or unacceptable in a given situation. Being a normative influence dimension, it is important to consider the source that promotes these prescriptions. For this reason, the injunctive norm was presented as communicated by different sources: the government, scientists, and an implicit source. The local descriptive norm refers to a behavior that is contextualized in situations that are close to the individual, as to increase the sense of belonging to a social group. A representative image of the source accompanied each norm, and each participant was assigned to one of the four following conditions (25% of the participants for each condition).

(1) Injunctive norm with a political source: “*The Government says that it is necessary to minimize verbal exchanges in indoor public places.”*
(2) Injunctive norm with a scientific source: “*Scientists say that it is necessary to minimize verbal exchanges in indoor public places.”*
(3) Injunctive norm with an implicit source: “*It is necessary to minimize verbal exchanges in indoor public places.”*
(4) Descriptive local norm: “*The inhabitants of your neighborhood have minimized verbal exchanges in indoor public places.”*

The participants were then asked to indicate, on a Likert scale ranging from 1 to 7, their answers to two different items: how much they agreed they should minimize verbal exchanges in indoor public places, and how much they intended to perform that behavior in the following days.

#### Manipulation 2: Content of the Message

The second objective of the study was to explore which type of content (i.e., neutral, emotional, exponential growth, or a combination of emotional and exponential growth) is most effective in promoting the adoption of infection prevention behaviors, such as social distancing, using personal protective equipment, and washing hands when the legislative restrictions are lifted.

**FIGURE 1:**
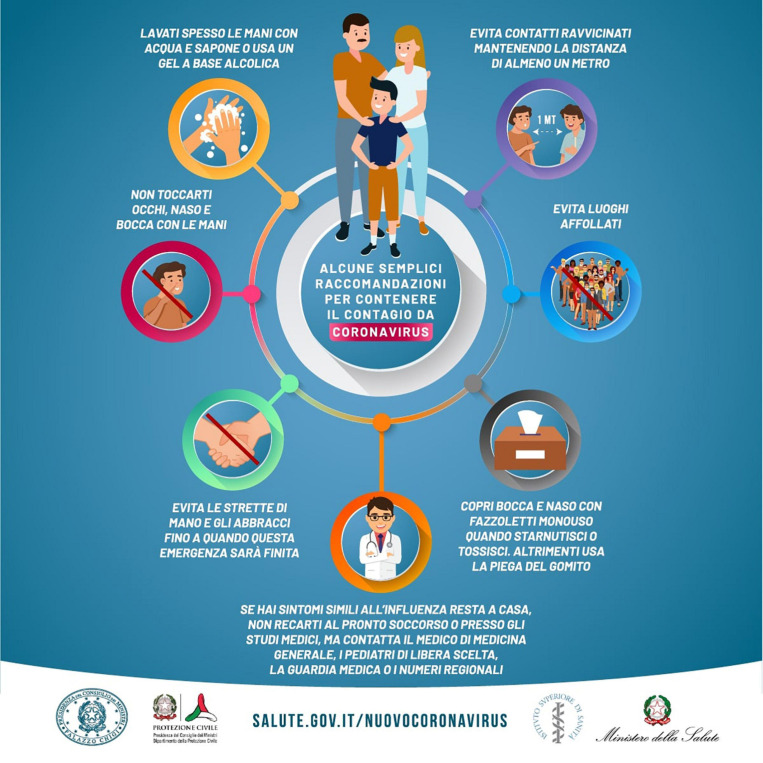
Poster from the Ministry of Health.

When investigating the effect of the content of the messages aimed at promoting preventive behavior during Phase 2, our study 1 and our study 2 were inspired by the research carried out by Lunn et al. (2020). However, since our research applies to the Italian context, it was necessary to make some changes to the experimental design. Vignettes were presented in the first person because the Italian Ministry of Health aimed to increase individual responsibility when there was no longer an obligation to stay at home. Moreover, we chose to use cartoons with drawings rather than real images to remain in line with the poster from the Ministry to which Italian citizens were exposed (Figure 1). We added one more cartoon that included overall emotion and exponential growth together. We hypothesize that by combining the two conditions, the intervention would be more effective: the exponential growth bias would be overcome, and the emotional aspect would increase the effectiveness of the stimuli.

**FIGURE 2:**
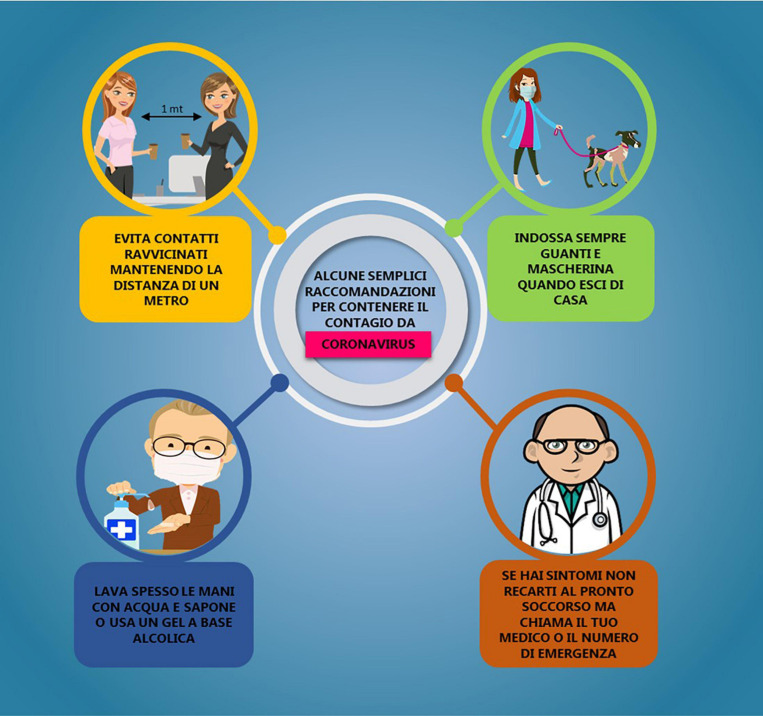
Control condition.

Each participant was assigned to one of the four conditions reported below (25% of the participants for each condition), in each of which, four preventive behaviors were communicated. The participants were then asked to indicate on a Likert scale ranging from 1 to 7 how much they were intended to adopt these preventive behaviors in the following days. Figure 1 shows a poster frequently used by the Italian Ministry of Health, and Figure 2 shows the control poster used in this study.

##### Condition 1

Control condition: Some simple recommendations to contain COVID-19 infection.

• Yellow message: *Avoid close contact by keeping a distance of one meter.*
• Green message: *Always wear a mask and gloves when you leave home.*
• Blue message: *Wash your hands often with soap and water or use an alcohol-based gel.*
• Brown message: *If you have symptoms, do not go to the emergency room but call your doctor or the emergency number.*

##### Condition 2

Emotion: You can save the people you care about from COVID-19.

• Yellow message: *So as not to infect colleagues, avoid close contact by keeping a distance of at least one meter even at work.*
• Green message: *Protect your friends from the virus, even in everyday activities outside the home, always wear gloves and a mask.*
• Blue message: *To keep your family safe, wash your hands often with soap and water, or use an alcohol-based gel.*
• Brown message: *To avoid endangering the health of patients at risk, do not go to the emergency room if you have symptoms but call your doctor or the emergency number.*

##### Condition 3

Exponential growth: Stop exponential growth of COVID-19 infection.

• Yellow message: *So as not to infect three people who will infect nine others, avoid close contact by keeping a distance of one meter.*
• Green message: *So as not to infect one individual, who in turn will infect others, always wear gloves and a mask when you leave the house.*
• Blue message: *To avoid passing the virus to four people who will pass it on to 16 others, wash your hands often with soap and water or use an alcohol-based gel.*
• Brown message: *To avoid endangering patients who will infect others, do not go to the emergency room if you have symptoms but call your doctor or the emergency number.*

##### Condition 4

Combination of emotion and exponential growth: Stop the exponential growth to save the people you care about from COVID-19.

• Yellow message: *So as not to infect three colleagues who will infect nine other people, avoid close contact by keeping a distance of one meter*.
• Green message: *So as not to infect a friend who will infect others, always wear gloves and a mask when you leave the house.*
• Blue message: *To avoid passing the virus to four people, including your family members, who will pass it on to 16 others, wash your hands often with soap and water or use an alcohol-based gel.*
• Brown message: *So as not to endanger the lives of patients, who will infect others, do not go to the emergency room if you have symptoms but call your doctor or the emergency number.*

### Results

#### Knowledge About the Behaviors Relevant to COVID-19 Infection Prevention

The knowledge of the participants on the relevant behaviors to contain the COVID-19 infection is in line with the provisions given by the Ministry of Health. The first three behaviors that participants of the national sample indicated, in order of importance, are social distancing (80.8% rated this behavior as the most important), the use of personal protective equipment (71.6% rated this behavior as the second most important), followed by washing hands (64%). The 24.8% of the participants rated staying at home and not to leave home for non-essential reasons among the three most important behaviors.

#### The Mental Representation of COVID-19

The mental representation of COVID-19 was mostly associated with disease (22%), contagion (20%), and negative emotions (16%), such as fear and worry. Only 1% of the participants referred to the economic consequences of the pandemic. When they are asked to choose from a list of words, they associated with the effects of COVID-19, the most commonly preferred terms were biological weapon (18.4%), flu (17.6%), and enclosure (15.8%), as negative associations; while in fourth place there was a positive association, solidarity (10%).

#### Essential and Not Essential Behaviors and Behavioral Intentions

During the lockdown, individual behaviors related to leaving home were regulated by the law. The allowed reasons to leave home included: going out to buy essential goods, going out to work, and going out to take care of relatives and neighbors in need. We refer to these when we talk about “essential behaviors.” When we refer to “non-essential” behaviors, actions such as going out to buy non-essential goods, meet people outside or at their home, go for a walk, or play sports, are considered. Most of these behaviors were prohibited during the lockdown or allowed with strong restrictions (e.g., it was possible, but not recommended, to work out or run, but this had to be within 200 meters from home).

The results demonstrate that the intention to implement “non-essential behaviors” increased in the post-lockdown phase compared to the behaviors implemented in the week before the administration of the questionnaire (*t*(399) = −13.483, *p* < 0.001), and this was as expected. This intention also increased significantly (*t*(393) = −15.332, *p* < 0.001) when referring to the return to ordinary life (defined as the moment from which the social distance was no longer necessary) compared to the phase following the lockdown. This meant that the participants realized that the end of the lockdown did not mean a return to ordinary life in terms of leaving home.

However, the participants who indicated “staying at home” and “avoiding going out for non-essential reasons” among the first three measures they considered necessary for the containment of COVID-19 infection, did not demonstrate different behaviors and different intentions than the other participants, [during the lockdown: *t*(134.243) = 0.005, *p* = 0.996; after the lockdown: *t*(134.780) = 0.348, *p* = 0.728]. However, the differences were significant between males and females. Males were more likely to perform non-essential behavior (*t*(398) = 3.359, *p* = 0.001), and more likely to do so in the future (*t*(398) = 4.515, *p* < 0.001).

#### Back to Normal Life

With regard to the estimated months before a return to ordinary life, the participants indicated a minimum of 1 month and a maximum of 48 months (mean = 8.53, SD = 6.068). This estimate was not statistically different between the age groups, gender, education, and geographical area (nor the area of infection).

#### Health Risk, Economic, Privacy, and Mobility

**FIGURE 3:**
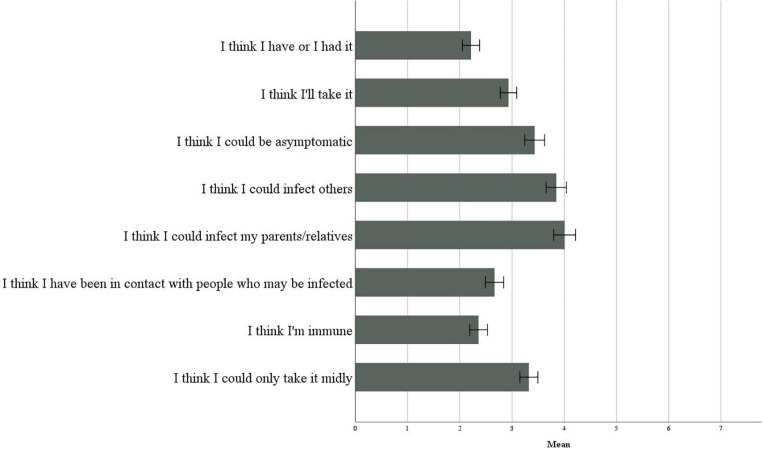
Perception of risk infection.

To assess how much the participants felt at risk of becoming infected with COVID-19, with reference to the risk of infecting themselves or infecting others, they were asked to indicate their degree of agreement to a series of statements on a Likert scale from 1 (completely disagree) to 7 (completely agree). Figure 3 illustrates the perception of risk infection.

We can observe that the averages of “I think I had it” and “I think I will get it” were significantly lower than “I think I could infect others” [respectively: *t*(399) = −14.624, *p* < 0.001; *t*(399) = −9.570, *p* < 0.001] and “I think I could infect my relatives and parents” [respectively: *t*(399) = −14.582, *p* < 0.001; *t*(399) = −10.651, *p* < 0.001]. The probability of getting the virus was perceived as very low, and there was a greater fear of infecting others rather than being infected, due to the 2 months of lockdown and the reduced contact with others that reinforced control over social relationships.

The results of the one-way ANOVA indicate that in the regions with high contagion, compared to all others, the participants tended to believe to a greater extent that they have or have had the virus (*F*(2) = 3.559, *p* = 0.029), they were asymptomatic (*F*(2) = 3.729, *p* = 0.025), they have been in contact with people who may be infected (*F*(2) = 6.667, *p* = 0.001), and that they may have had the virus slightly (*F*(2) = 4.811, *p* = 0.009). This difference also exists for the possibility of infecting others (*F*(2) = 3.230, *p* = 0.041); however, there were no significant differences between infection regions in the possibility of infecting parents or relatives (*F*(2) = 0.027, *p* = 0.974). Between the age groups, there was no difference in “I think I am immune” (*F*(3) = 0.873, *p* = 0.455) and “I think I could contract it in a mild form” (*F*(3) = 0.760, *p* = 0.517).

A general risk perception score was created from the following variables (appropriately oriented): the negative representation of COVID-19 infection, non-essential behavior during the lockdown, and after the lockdown, return to ordinary life assessment, health risk perception, economic risk perception, privacy risk perception, and mobility risk perception. The health risk perception was generated from the four items (1) I think I will get it, (2) I think I could infect others, (3) I think I could infect relatives and parents, and (4) I think I could contract it only in a mild form. To assess the economic risk (related to work), the privacy risk (related to the contact tracing app), and the risk associated with mobility, the participants were asked to respond to several statements with a degree of agreement (Likert 1–7). Three levels of risk perception (low, medium, and high) were created, starting with the quartile division. The first quartile corresponds to low-risk perception, the fourth quartile to high-risk perception, and the central quartiles to medium-risk perception. Table 2 shows the descriptive information related to health, economic, mobility, and privacy risk perception.

**TABLE 2:** Risk perception descriptions.

	Health risk perception	Economic risk perception	Mobility risk perception	Privacy risk perception
Mean	4.22	4.59	4.25	3.74
SD	1.033	1.111	1.095	0.856
α_*Chro*_	0.710	0.756	0.820	0.658

#### Trust in Sources

With regard to the trust in sources of communication and emergency management, Table 3 summarizes the sample trust assessments for each source. The scientific community was trusted more than the Government (*p* < 0.001), and the two measures correlate positively with each other (*r* = 0.588, *p* < 0.001).

**TABLE 3:** Level of trust in various sources of communication.

	Mean	SD
Scientific community	5.20	1.64
Government	3.96	1.93

**FIGURE 4:**
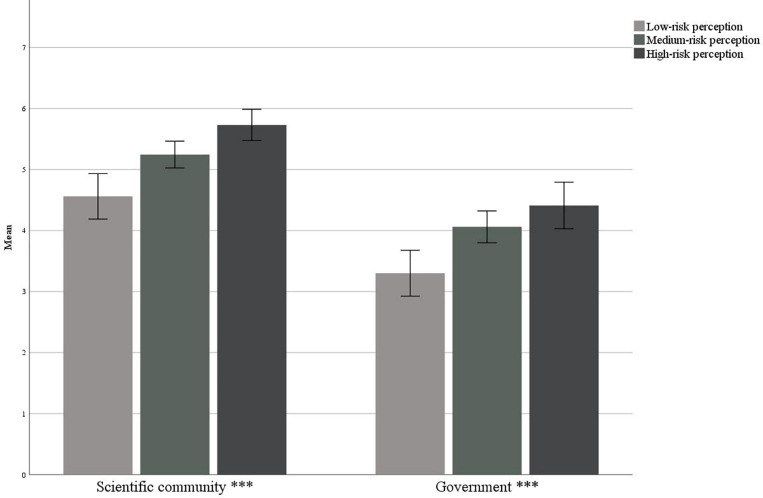
Trust in sources in relation to the risk perception ^∗∗∗^*p* < 0.001.

**FIGURE 5:**
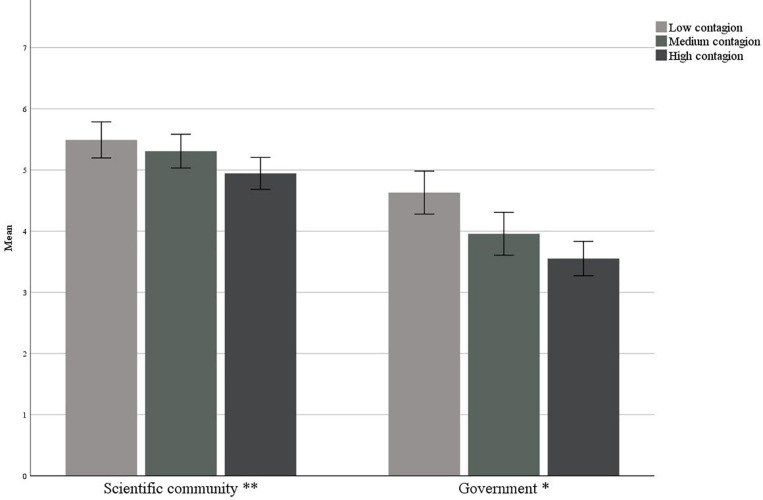
Trust in sources in relation to the contagion area ^∗^*p* < 0.05 ^∗∗^*p* < 0.005.

In Figure 4, we can observe that in the national sample, the confidence in the Government and scientific community as sources of communication and emergency management was statistically different between participants who have low-, medium-, and high-risk perceptions. In particular, as perceived risk increased, confidence in both the scientific community and the Government increased. Considering the areas of contagion instead, we can observe that in the areas of greater contagion, there was a lowering of confidence (Figure 5).

#### Manipulation 1: Norms and Sources

Two models of ordinal logistic regression were adopted; one for the degree of agreement and one for the behavioral intention. The measures of the degree of agreement and behavioral intention were classified (as in Lunn et al., 2020) as low (≤5), medium (6), and high (7). The main effects of the experimental conditions and risk perception were considered as the predictors in the models. We take account of the interaction between these two variables. The models included confidence in the Government and the scientific community as covariates. We also considered demographic controls (gender, age range, and contagion area) and their interaction with the conditions.

In relation to the agreement with the behavioral norm, 40.0% of the respondents were in the low range, 22.0% were in the medium range, and 38.0% were in the high range. The prediction model demonstrated goodness of fit to our observed data (χ^2^ (37) = 86.481, *p* < 0.001). High levels of agreement were associated with a higher trust in the Government (but a significant effect of trust in the scientific community did not emerge). Low and medium risk perception were associated with a lower level of agreement. The main effect of experimental conditions and the interaction with risk perception, age and contagion area, did not affect the agreement (Table 4).

**TABLE 4:** Ordinal logistic regressions for agreement with the behavioral norm (manipulation 1).

	Manipulation 1: Agreement with the Behavioral Norm						
		Estimate	SE	OR (95%CI)		EXP(b)	*p*
**Predictors**							
Exp. Cond	Inj_Gov	1.460	1.069	−0.636	3.555	1.864	0.172
	Inj_Scient	0.274	0.947	−1.583	2.130	0.083	0.773
	Descr	0.251	0.899	−1.512	2.013	0.078	0.781
	Impl	0(ref)					
Risk Percep	Low	−1.384	0.600	−2.560	−0.207	5.313	0.021
	Medium	−1.486	0.519	−2.504	−0.468	8.185	0.004
	High	0(ref)					
**Covariates**							
Trust_Scient		0.157	0.081	−0.002	0.316	3.728	0.054
Trust_Gov		0.151	0.067	0.019	0.282	5.048	0.025
**Demographics**							
Gender	Male	1.023	0.425	0.189	1.856	5.787	0.016
	Female	0(ref)					
Age range	18–30	−0.043	0.673	−1.363	1.276	0.004	0.949
	31–45	−0.419	0.627	−1.648	0.811	0.446	0.504
	46–60	−0.203	0.601	−1.380	0.974	0.115	0.735
	61–70	0(ref)					
Contagion area	Low	0.499	0.541	−0.561	1.558	0.852	0.356
	Medium	0.366	0.491	−0.595	1.328	0.558	0.455
	High	0(ref)					
**Interactions**							
**Exp. Conditions × Risk Perception**							
	Inj_Gov × Low	−0.390	0.869	−2.092	1.312	0.202	0.653
	Inj_Gov × Medium	0.164	0.761	−1.328	1.656	0.046	0.830
	Inj_Gov × High	0(ref)					
	Inj_Scient × Low	0.433	0.833	−1.200	2.066	0.270	0.603
	Inj_Scient × Medium	1.455	0.710	0.064	2.846	4.202	0.040
	Inj_Scient × High	0(ref)					
	Descr × Low	0.909	0.828	−0.714	2.532	1.205	0.272
	Descr × Medium	0.655	0.714	−0.745	2.055	0.841	0.359
	Descr × High	0(ref)					
	Impl × Low	0(ref)					
	Impl × Medium	0(ref)					
	Impl × High	0(ref)					
**Exp. Conditions × Gender**							
	Inj_Gov × Male	−1.023	0.598	−2.194	0.148	2.935	0.087
	Inj_Gov × Female	0(ref)					
	Inj_Scient × Male	−1.578	0.594	−2.742	−0.414	7.057	0.008
	Inj_Scient × Female	0(ref)					
	Descr × Male	−1.096	0.583	−2.238	0.045	3.542	0.060
	Descr × Female	0(ref)					
	Impl × Male	0(ref)					
	Impl × Female	0(ref)					
**Exp. Conditions × Age range**							
	Inj_Gov × 18–30	−0.958	1.021	−2.958	1.042	0.881	0.348
	Inj_Gov × 31–45	−0.167	0.902	−1.935	1.601	0.034	0.853
	Inj_Gov × 46–60	−0.518	0.894	−2.270	1.235	0.335	0.563
	Inj_Gov × 61–70	0(ref)					
	Inj_Scient × 18–30	−0.885	1.034	−2.911	1.142	0.732	0.392
	Inj_Scient × 31–45	−0.144	0.862	−1.834	1.546	0.028	0.867
	Inj_Scient × 46–60	−0.872	0.864	−2.566	0.821	1.019	0.313
	Inj_Scient × 61–70	0(ref)					
	Descr × 18–30	−0.647	0.998	−2.603	1.310	0.420	0.517
	Descr × 31–45	−0.172	0.876	−1.889	1.545	0.038	0.845
	Descr × 46–60	0.666	0.864	−1.028	2.360	0.593	0.441
	Descr × 61–70	0(ref)					
	Impl × 18–30	0(ref)					
	Impl × 31–45	0(ref)					
	Impl × 46–60	0(ref)					
	Impl × 61–70	0(ref)					
**Exp. Conditions × Contagion area**							
	Inj_Gov × Low	−0.589	0.701	−1.962	0.784	0.707	0.401
	Inj_Gov × Medium	−0.307	0.726	−1.729	1.115	0.179	0.672
	Inj_Gov × High	0(ref)					
	Inj_Scient × Low	0.809	0.743	−0.648	2.266	1.185	0.276
	Inj_Scient × Medium	−0.131	0.697	−1.497	1.236	0.035	0.851
	Inj_Scient × High	0(ref)					
	Descr × Low	−0.813	0.758	−2.299	0.673	1.149	0.284
	Descr × Medium	−0.251	0.665	−1.555	1.053	0.142	0.706
	Descr × High	0(ref)					
	Impl × Low	0(ref)					
	Impl × Medium	0(ref)					
	Impl × High	0(ref)					

In relation to the behavioral intention, 48.0% of the respondents were in the low range, 17.3% were in the medium range, and 34.8% were in the high range. The prediction model demonstrated goodness of fit to our observed data (χ^2^ (37) = 75.626, *p* < 0.001). High levels of behavioral intention were associated with the injunctive norm communicated by the Government, but this condition is less effective for those with a medium risk perception. The low-risk perception was associated with low intentions, and in this case, trust in the Government did not have an effect. Demographics and their interactions with conditions did not affect the intention (Table 5).

**TABLE 5:** Ordinal logistic regressions for the behavioral intention (manipulation 1).

	Manipulation 1: Intention						
		Estimate	SE	OR (95%CI)		EXP(b)	*p*
**Predictors**							
Exp. Cond	Inj_Gov	2.333	1.0998	0.177	4.488	4.499	0.034
	Inj_Scient	0.248	0.9407	−1.6	2.091	0.069	0.792
	Descr	0.901	0.9189	−0.9	2.702	0.962	0.327
	Impl	0(ref)					
Risk Percep	Low	−1.269	0.6422	−2.53	−0.01	3.904	0.048
	Medium	−0.514	0.4754	−1.45	0.418	1.167	0.28
	High	0(ref)					
**Covariates**							
Trust_Scient		0.134	0.0823	−0.03	0.295	2.644	0.104
Trust_Gov		0.002	0.0695	−0.14	0.138	0	0.982
**Demographics**							
Gender	Male	0.124	0.4267	−0.71	0.961	0.085	0.771
	Female	0(ref)					
Age range	18–30	−0.724	0.7015	−2.1	0.651	1.066	0.302
	31–45	0.194	0.6198	−1.02	1.409	0.098	0.754
	46–60	−0.709	0.6078	−1.9	0.482	1.362	0.243
	61–70	0(ref)					
Contagion area	Low	−0.543	0.5489	−1.62	0.533	0.977	0.323
	Medium	−0.213	0.501	−1.2	0.769	0.18	0.671
	High	0(ref)					
**Interactions**							
**Exp. Conditions × Risk Perception**							
	Inj_Gov × Low	−1.233	0.952	−3.100	0.634	1.676	0.195
	Inj_Gov × Medium	−1.763	0.792	−3.315	−0.210	4.950	0.026
	Inj_Gov × High	0(ref)					
	Inj_Scient × Low	−0.131	0.869	−1.835	1.572	0.023	0.880
	Inj_Scient × Medium	0.026	0.671	−1.290	1.342	0.001	0.969
	Inj_Scient × High	0(ref)					
	Descr × Low	0.023	0.867	−1.676	1.723	0.001	0.978
	Descr × Medium	−0.146	0.677	−1.472	1.181	0.046	0.830
	Descr × High	0(ref)					
	Impl × Low	0(ref)					
	Impl × Medium	0(ref)					
	Impl × High	0(ref)					
**Exp. Conditions × Gender**							
	Inj_Gov × Male	−0.386	0.628	−1.617	0.846	0.377	0.539
	Inj_Gov × Female	0(ref)					
	Inj_Scient × Male	−0.449	0.583	−1.592	0.694	0.592	0.442
	Inj_Scient × Female	0(ref)					
	Descr × Male	−0.580	0.595	−1.746	0.587	0.949	0.330
	Descr × Female	0(ref)					
	Impl × Male	0(ref)					
	Impl × Female	0(ref)					
**Exp. Conditions × Age range**							
	Inj_Gov × 18–30	−0.624	1.055	−2.692	1.445	0.349	0.554
	Inj_Gov × 31–45	−0.776	0.872	−2.485	0.932	0.793	0.373
	Inj_Gov × 46–60	−0.859	0.914	−2.651	0.932	0.884	0.347
	Inj_Gov × 61–70	0(ref)					
	Inj_Scient × 18–30	−0.245	1.042	−2.288	1.797	0.055	0.814
	Inj_Scient × 31–45	−0.145	0.846	−1.803	1.513	0.029	0.864
	Inj_Scient × 46–60	0.962	0.854	−0.712	2.636	1.269	0.260
	Inj_Scient × 61–70	0(ref)					
	Descr × 18–30	0.230	1.017	−1.763	2.222	0.051	0.821
	Descr × 31–45	−0.899	0.869	−2.602	0.804	1.070	0.301
	Descr × 46–60	0.871	0.873	−0.840	2.581	0.995	0.319
	Descr × 61–70	0(ref)					
	Impl × 18–30	0(ref)					
	Impl × 31–45	0(ref)					
	Impl × 46–60	0(ref)					
	Impl × 61–70	0(ref)					
**Exp. Conditions × Contagion area**							
	Inj_Gov × Low	0.724	0.722	−0.692	2.140	1.004	0.316
	Inj_Gov × Medium	−0.308	0.776	−1.829	1.213	0.158	0.691
	Inj_Gov × High	0(ref)					
	Inj_Scient × Low	0.598	0.729	−0.832	2.028	0.672	0.412
	Inj_Scient × Medium	0.860	0.700	−0.511	2.232	1.512	0.219
	Inj_Scient × High	0(ref)					
	Descr × Low	0.864	0.765	−0.635	2.362	1.277	0.258
	Descr × Medium	−0.081	0.687	−1.427	1.265	0.014	0.906
	Descr × High	0(ref)					
	Impl × Low	0(ref)					
	Impl × Medium	0(ref)					
	Impl × High	0(ref)					

#### Manipulation 2: Content of the Message

The memorability and effectiveness of the experimental conditions used were determined and two models of ordinary logistic regression were performed. The measures of memorability and effectiveness were classified (as in Lunn et al., 2020) as low (≤5), medium (6), and high (7). Knowledge of the prevention behaviors indicated by the Ministry of Health was included in the models.

The neutral condition was associated with a higher memorability (EXP(B) = 7.840, *p* = 0.005). However, no condition was higher in effectiveness than the others. Descriptions regarding memorability, effectiveness, and intentions are reported in Table 6.

**TABLE 6:** Memorability, effectiveness, and intention descriptions.

	Memorability	Effectiveness	Intention
Low	14.2%	17.3%	28.0%
Medium	37.3%	42.5%	30.8%
High	49.5%	40.2%**	41.3%
Goodness of fit	17.501**	15.915**	46.142***
Nagelkerke	0.050	0.045	0.123

***p < 0.001, **p < 0.005.

To establish which condition was more effective in promoting intention toward prevention behaviors, an ordinal logistic regression model was performed. The main effects of the experimental conditions and risk perception were considered as the predictors in the models. We take account of the interaction between these two variables. We also consider a measure of personal knowledge about the main prevention behaviors, demographic controls (gender, age range, and contagion area) and their interaction with the conditions.

In relation to intention, 28.0% of the respondents were in the low range, 30.8% were in the medium range, and 41.3% were in the high range. The prediction model demonstrated goodness of fit to our observed data (χ^2^ (38) = 80.223, *p* < 0.001).

The behavioral intention was associated with perceived risk and with the knowledge of prevention behaviors. As reported in Table 7, for those with medium risk perception, the exponential growth condition was more effective in promoting behavioral intention. Demographics and their interactions with conditions did not affect the intention.

**TABLE 7:** Ordinal logistic regressions for the behavioral intention (manipulation 2).

	Manipulation 2: behavioral Intention						
		Estimate	SE	OR (95%CI)		EXP(b)	*p*
**Predictors**							
Exp. Cond	Neutral	0.983	1.199	−1.366	3.332	0.673	0.412
	Emotional	−1.482	1.071	−3.582	0.617	1.916	0.166
	Exp. Growth	−1.103	1.051	−3.162	0.956	1.103	0.294
	Combined	0(ref)					
Risk Percep	Low	−1.170	0.599	−2.344	0.004	3.816	0.051
	Medium	−1.209	0.529	−2.245	−0.172	5.224	0.022
	High	0(ref)					
Knowledge	None	−0.483	1.092	−2.624	1.658	0.196	0.658
	Min	−0.809	0.609	−2.003	0.384	1.766	0.184
	Med	−0.426	0.445	−1.298	0.447	0.914	0.339
	Max	0(ref)					
**Demographics**							
Gender	Male	−0.503	0.420	−1.327	0.321	1.433	0.231
	Female	0(ref)					
Age range	18–30	−0.682	0.723	−2.099	0.735	0.889	0.346
	31–45	−0.310	0.646	−1.576	0.956	0.230	0.631
	46–60	−0.335	0.620	−1.551	0.881	0.291	0.589
	61–70	0(ref)					
Contagion area	Low	−0.539	0.507	−1.534	0.456	1.128	0.288
	Medium	−0.052	0.499	−1.031	0.927	0.011	0.917
	High	0(ref)					
**Interactions**							
**Exp. Conditions × Risk Perception**							
	Neutral × Low	−0.910	0.934	−2.740	0.919	0.951	0.329
	Neutral × Medium	−0.227	0.830	−1.853	1.399	0.075	0.785
	Neutral × High	0(ref)					
	Emotional × Low	−0.008	0.845	−1.664	1.648	0.000	0.992
	Emotional × Medium	0.681	0.719	−0.729	2.091	0.896	0.344
	Emotional × High	0(ref)					
	Exp. G. × Low	0.928	0.851	−0.740	2.596	1.188	0.276
	Exp. G. × Medium	1.527	0.718	0.120	2.934	4.523	0.033
	Exp. G. × High	0(ref)					
	Comb × Low	0(ref)					
	Comb × Medium	0(ref)					
	Comb × High	0(ref)					
**Exp. Conditions × Knowledge**							
	Neutral × None	−0.406	1.4562	−3.26	2.448	0.078	0.781
	Neutral × Min	−0.374	0.8843	−2.107	1.36	0.179	0.673
	Neutral × Med	−0.154	0.6748	−1.477	1.168	0.052	0.819
	Neutral × Max	0(ref)					
	Emotional × None	−21.151	18738.2	−36747	36705	0.000	0.999
	Emotional × Min	−0.842	0.8776	−2.562	0.878	0.921	0.337
	Emotional × Med	0.291	0.6327	−0.949	1.531	0.211	0.646
	Emotional × Max	0(ref)					
	Exp. G. × None	−0.26	1.3599	−2.926	2.405	0.037	0.848
	Exp. G. × Min	0.194	0.871	−1.513	1.901	0.05	0.824
	Exp. G. × Med	0.768	0.6348	−0.476	2.012	1.464	0.226
	Exp. G. × Max						
	Comb × None	0(ref)					
	Comb × Min	0(ref)					
	Comb × Med						
	Comb × Max	0(ref)					
**Exp. Conditions × Gender**							
	Neutral × Male	−0.123	0.605	−1.308	1.063	0.041	0.839
	Neutral × Female	0(ref)					
	Emotional × Male	0.009	0.589	−1.146	1.164	0.000	0.987
	Emotional × Female	0(ref)					
	Exp. G. × Male	0.168	0.576	−0.961	1.297	0.085	0.771
	Exp. G. × Female	0(ref)					
	Comb × Male	0(ref)					
	Comb × Female	0(ref)					
**Exp. Conditions × Age range**							
	Neutral × 18–30	−1.007	1.055	−3.074	1.060	0.912	0.340
	Neutral × 31–45	−0.735	0.893	−2.485	1.015	0.678	0.410
	Neutral × 46–60	−0.151	0.901	−1.918	1.615	0.028	0.867
	Neutral × 61–70	0(ref)					
	Emotional × 18–30	−0.107	1.054	−2.173	1.958	0.010	0.919
	Emotional × 31–45	0.565	0.880	−1.160	2.291	0.412	0.521
	Emotional × 46–60	0.686	0.865	−1.009	2.382	0.630	0.427
	Emotional × 61–70	0(ref)					
	Exp. G. × 18–30	−0.566	1.036	−2.595	1.464	0.298	0.585
	Exp. G. × 31–45	−1.085	0.922	−2.893	0.722	1.385	0.239
	Exp. G. × 46–60	0.056	0.925	−1.758	1.869	0.004	0.952
	Exp. G. × 61–70	0(ref)					
	Comb × 18–30	0(ref)					
	Comb × 31–45	0(ref)					
	Comb × 46–60	0(ref)					
	Comb × 61–70	0(ref)					
**Exp. Conditions × Contagion area**							
	Neutral × Low	0.261	0.686	−1.084	1.606	0.144	0.704
	Neutral × Medium	−0.209	0.759	−1.697	1.279	0.076	0.783
	Neutral × High	0(ref)					
	Emotional × Low	0.154	0.728	−1.274	1.581	0.045	0.833
	Emotional × Medium	0.955	0.699	−0.414	2.325	1.869	0.172
	Emotional × High	0(ref)					
	Exp. G. × Low	0.706	0.741	−0.747	2.158	0.907	0.341
	Exp. G. × Medium	−0.090	0.682	−1.426	1.246	0.017	0.895
	Exp. G. × High	0(ref)					
	Comb × Low	0(ref)					
	Comb × Medium	0(ref)					
	Comb × High	0(ref)					

Further ordinal logistic regression models were conducted, without modifying the dependent variables and reported in Appendix (see Supplementary Material).

## Study 2

Study 2 focuses on a sample of undergraduate students of Milano-Bicocca University in order to investigate, through our first manipulation, if the University as an information source, could play a role in promoting preventive behavior, in addition to the Government. The message presented to participants was to “limit leaving home to the minimum required in phase 2,” since, despite the legislative norms, young people tended to go out of home for avoidable reasons in Milan in this period^1^. As for study 1, the second manipulation focuses on identifying which aspects between emotional, exponential growth, both, or neutral, are most effective in promoting contagion prevention behavior.

### Methods

#### Participants

One hundred sixty-five undergraduate students of the University of Milano-Bicocca University (females = 116, aged between 19 and 60, mean = 23.90, SD = 5.404) took part in the experiment. Most were residents in Lombardy, which was a region of high contagion during the lockdown. Students received a training credit for undertaking the 20-min online study. A further 31 subjects (16%) were excluded from the analysis because they claimed to have contracted COVID-19 or were in close contact with people who had contracted it. Study 1 and study 2 consider two different groups of participants for two reasons in particular. As the same rules may not have been perceived in the same way by people of different ages, we wanted to investigate two different target groups separately (also proposing different rules). In particular, this was because the group of students is most likely to perform risky contagion behavior as they have more active social lives and are more likely to participate in assemblages. For this reason, we submitted a message to the students focusing on limiting how often they leave their homes, while the message to the national sample was based on minimizing verbal exchanges in indoor public places. Moreover, through the first manipulation, we expected the two samples to have different confidence in the sources that promote the behaviors; we anticipated that we would be able to identify a source very close to the sample of students (university), which cannot be done with a national sample in general. We also choose to test a students’ sample to explore with the second manipulation if messages focusing on different aspects would influence both groups differently. Using a national sample, moreover, allowed us to investigate whether there were differences in the different regions depending on the level of contagion in the perceived trust of sources.

#### Materials and Design

As with study 1, the survey data were collected through a self-administered questionnaire accessible through an online platform. The survey was administered to the students’ sample from May 1 (the last week of the lockdown) to June 8. The questionnaire was structured as in Study 1, and composed of the same 6 parts, except for what concerns risk perception (see Appendix 2): in this case, since we refer to students, instead of the economic risk perception we investigated the academic risk perception^2^.

The participants were also subjected to two experimental manipulations, and each subject was randomly shown one of four conditions for each manipulation. The first manipulation differed from Study 1 for the preventive behavior suggested and for the sources that communicate it, while the second manipulation was identical.

#### Manipulation 1: Norms and Sources

The first manipulation of Study 2 consisted in the same of study 1: we aimed at identifying the best norm and source in promoting preventive behavioral intention, taking into account the confidence in the messenger. In particular, we decided to test a sample of students to investigate with the first manipulation, if the University (as the source of the message) could play a role in promoting preventive behavior, in addition to the Government. The behavior we aimed to promote was in this case, to “limit leaving home to the minimum required in phase 2.” We believed that this was the key behavior to reinforce in order to promote prevention, since during the lockdown in Italy, and especially in the center of Milan, despite legislative restrictions, many young people went out of their homes for non-essential reasons Two different types of norms were taken into consideration: the *injunctive norm* (“*it is necessary to*”) and the *descriptive norm* (*“the majority of students from Lombardy, including those at Bicocca-University, intend to limit [*…*]”*). The injunctive and descriptive norms were presented as communicated by different sources: the Government and the University of Milano-Bicocca. An image representative of the source accompanied each norm, and each participant was assigned to one of the four conditions resulting from varying the two sources and two norm conditions’ (approximately 25% of the participants for each condition):

(1) Injunctive norm with a political source: “*The Government says that it is necessary to limit leaving home to the minimum necessary in phase 2”*;
(2) Injunctive norm with a university source: *“The University of Milano-Bicocca says that it is necessary to limit leaving home to the minimum necessary in phase 2”;*
(3) Descriptive norm with a political source: “*The* Government *says that the majority of students from Lombardy (including those at Bicocca-University) intend to limit leaving home to the minimum necessary in phase 2”;*
(4) Descriptive norm with a university source: “*The University of Milano-Bicocca says that the majority of students from Lombardy (including those at Bicocca-University) intend to limit leaving home to the minimum necessary in phase 2”;*

The participants were asked to indicate, from 1 to 7 Likert scales, how much they agreed to adopt this preventive behavior in phase 2.

#### Manipulation 2: Content of the Message

As with study 1, we also wanted to explore which type of content is for students most effective in promoting the adoption of infection prevention behaviors. The students’ sample received the same materials of the national sample. As previously each participant was assigned to one of the four conditions (approximately between 20 and 30% of the participants for each condition) reported in Study 1 (Emotion, Exponential Growth, Combination of emotion and exponential growth and control condition). The participants were then asked to indicate, from 1 to 7 Likert scales, how much they were intended to adopt these preventive behaviors in the following days.

### Results

#### Knowledge About the Behaviors Relevant to COVID-19 Infection Prevention

The knowledge of the participants on the relevant behaviors to contain COVID-19 infection seems to be in line with the provisions given by the Ministry of Health. In particular, 81.2% of the subjects indicated they had socially distanced themselves, 74.5% used personal protective equipment, and 64.8% had implemented handwashing and surface hygiene behaviors. Staying at home and not going out for non-essential reasons was considered by the 22.4% of participants within the first three relevant behaviors. These results are similar to those obtained from the national sample.

#### The Mental Representation of COVID-19

The mental representation of COVID-19 is mostly associated with contagion (17%), illness (16%), and negative emotions (15%) such as fear and worry. No participants referred to the economic consequences of the pandemic. When the participants were asked to choose from a list of words that they associated with the effects of COVID-19, the results illustrate that the preferred terms are flu (22.5%), enclosure (21.9%), and solidarity (16.2%).

#### Essential and Not Essential Behaviors and Behavioral Intentions

As with Study 1, we refer to “essential behaviors” when we talk about the allowed reasons to leave home regulated by the law, which are opposed to the “non-essential” behaviors, which were mostly prohibited during the lockdown or allowed with strong restrictions. The results showed that, as expected, the intention to implement “non-essential behaviors” increased in the post-lockdown phase compared to the behaviors implemented in the week before the administration of the questionnaire (*t*(164) = −10.874, *p* < 0.001). This intention also increased significantly (*t*(156) = −14.557, *p* < 0.001) when referring to the return to ordinary life compared to the post-lockdown phase. The participants who indicated “staying at home” and “avoiding going out for non-essential reasons” among the first three necessary measures for the containment of the contagion, did not demonstrate different behaviors or intentions than the others [during the lockdown: *t*(68.087) = 0.683, *p* = 0.497; after the lockdown: *t*(66.014) = 1.657, *p* = 0.102].

#### Back to Normal Life

The students estimated a return to normal life from a minimum of 1 month and a maximum of 24 months (mean = 7.20, SD = 4.229). This estimate was not statistically different across the genders (*t*(62.232) = −0.389, *p* = 0.699).

#### Health Risk, Academic Risk, Privacy, and Mobility

**FIGURE 6:**
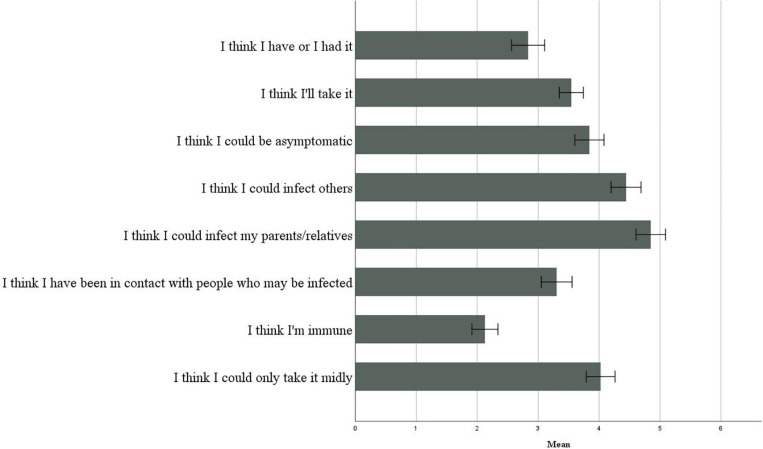
Perception of risk infection.

As with Study 1, participants were asked to indicate their degree of agreement to a series of statements on a Likert scale from 1 (completely disagree) to 7 (completely agree). Figure 6 illustrates the perception of risk infection. Results showed that the averages of “I think I had it” and “I think I will get it” were significantly lower than “I think I could infect others” [respectively: *t*(159) = −9.962, *p* < 0.001; *t*(158) = −6.450, *p* < 0.001] and “I think I could infect my relatives and parents” [respectively: *t*(161) = −13.631, *p* < 0.001; *t*(160) = −10.025, *p* < 0.001].

The general risk perception score was created from the same risk perception scales taken into consideration in Study 1, except for the economic risk perception which was replaced by the academic risk perception (related to the academic career). The items used are reported in the Appendix. Table 8 illustrates the descriptive information related to health, academic, mobility, and privacy risk perception.

**TABLE 8:** Risk perception descriptions.

	Health risk perception	Academic risk perception	Mobility risk perception	Privacy risk perception
Mean	4.33	3.36	4.56	3.83
SD	0.767	0.989	0.804	1.016
α_*Chro*_	0.550	0.664	0.862	0.830

#### Trust in Sources

**FIGURE 7:**
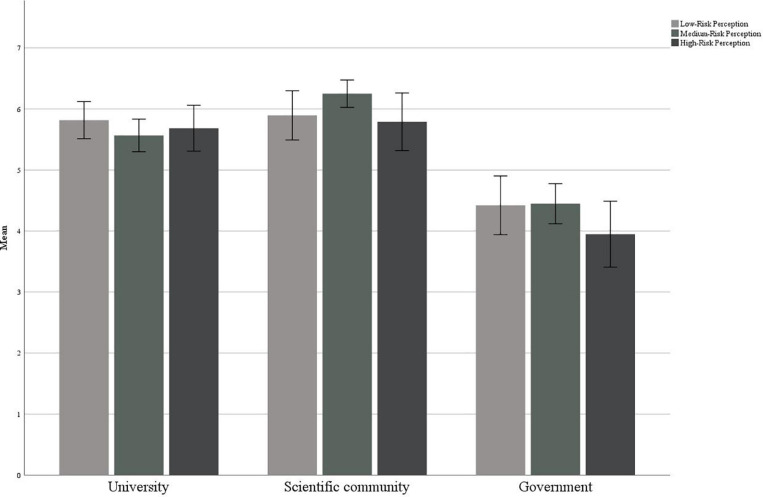
Trust in sources in relation to the perception of risk.

In Table 9 the sample’s trust in each source of communication is reported. As results show, the confidence in the scientific community was significantly higher than in the Government (*t*(160) = 14.607, *p* < 0.001), with a positive correlation (*r* = 0.399, *p* < 0.001), and then in the university sources (*t*(160) = 3.656, *p* < 0.001), with a positive correlation (*r* = 0.310, *p* < 0.001). Moreover, the trust in the university sources was higher than for the Government sources (*t*(160) = 9.742, *p* < 0.001). The two measures correlate positively but weakly (*r* = 0.157, *p* = 0.047). There were no differences in trust in relation to risk perception (Figure 7).

**TABLE 9:** Level of trust in various sources of communication.

	Mean	SD
Scientific community	6.02	1.21
Government	4.29	1.45
University	5.62	1.14

#### Manipulation 1: Norms and Sources

The degree of agreement was classified (as in Lunn et al., 2020) as low (≤5), medium (6), and high (7). The main effects of the experimental conditions and risk perception were considered as the predictors in the models. We take account of the interaction between these two variables. The models included confidence in the Government and the scientific community as covariates. We also considered demographic controls, but only for gender (age range and contagion area were excluded because there was no variability: 95.5% of the sample belongs to the age range 18–30; 87.2% of the participants belongs to the high contagion area) and its interactions with the conditions.

In relation to the behavioral intention, 27.6% of the respondents were in the low range, 30.9% were in the medium range, and 41.4% were in the high range. The prediction model demonstrated goodness of fit to our observed data (χ^2^ (17) = 52.001, *p* < 0.001).

Low levels of behavioral intention were associated with the descriptive norm communicated by the Government, and low and medium risk perception was associated with a lower intention. Among the participants who had a low-risk perception compared to those who had medium-risk/high-risk, the injunctive and descriptive norms of the Government, and the descriptive norm promoted by the university, seemed to be more influential. Gender and its interactions with conditions did not affect the intention. Additionally, the confidence in the Government had an effect, while confidence in the scientific community did not (Table 10).

**TABLE 10:** Ordinal logistic regressions for the behavioral intention in the student sample (manipulation 1).

	Manipulation 1: Intention						
		Estimate	SE	OR (95%CI)		EXP(b)	*p*
**Predictors**							
Exp. Cond	Descr_Gov	−3.182	1.294	−5.718	−0.646	6.049	0.014
	Descr_Univ	−1.570	1.315	−4.147	1.007	1.425	0.233
	Inj_Gov	−1.219	1.333	−3.831	1.394	0.836	0.361
	Inj_Univ	0(ref)					
Risk Percep	Low	−5.863	1.426	−8.657	−3.068	16.904	0.000
	Medium	−2.640	1.209	−5.009	−0.271	4.771	0.029
	High	0(ref)					
**Covariates**							
Trust_Univ		0.065	0.158	−0.244	0.375	0.172	0.678
Trust_Gov		0.294	0.124	0.050	0.538	5.592	0.018
**Demographics**							
Gender	Male	0.686	0.933	−1.142	2.514	0.541	0.462
	Female	0(ref)					
**Interactions**							
**Exp. Conditions × Risk Perception**							
	Descr_Gov × Low	4.786	1.673	1.508	8.065	8.187	0.004
	Descr_Gov × Medium	2.187	1.442	−0.639	5.014	2.301	0.129
	Descr_Gov × High	0(ref)					
	Descr_Univ × Low	3.518	1.713	0.161	6.874	4.219	0.040
	Descr_Univ × Medium	1.841	1.471	−1.042	4.724	1.566	0.211
	Descr_Univ × High	0(ref)					
	Inj_Gov × Low	3.722	1.755	0.282	7.161	4.498	0.034
	Inj_Gov × Medium	1.457	1.426	−1.338	4.252	1.044	0.307
	Inj_Gov × High	0(ref)					
	Inj_Univ × Low	0(ref)					
	Inj_Univ × Medium	0(ref)					
	Inj_Univ × High	0(ref)					
**Exp. Conditions × Gender**							
	Descr_Gov × Male	−0.261	1.313	−2.835	2.312	0.040	0.842
	Descr_Gov × Female	0(ref)					
	Descr_Univ × Male	−1.269	1.200	−3.621	1.083	1.118	0.290
	Descr_Univ × Female	0(ref)					
	Inj_Gov × Male	−1.535	1.145	−3.779	0.709	1.798	0.180
	Inj_Gov × Female	0(ref)					
	Inj_Univ × Male	0(ref)					
	Inj_Univ × Female	0(ref)					

#### Manipulation 2: Content of the Message

As with Study 1, the memorability and effectiveness of the experimental conditions used were determined and two models of ordinary logistic regression were performed. The measures of memorability and effectiveness were classified (as in Lunn et al., 2020) as low (≤5), medium (6), and high (7). Knowledge of the prevention behaviors indicated by the Ministry of Health was included in the models.

The neutral condition was associated with a higher memorability (EXP(B) = 6.630, *p* = 0.010), and with higher effectiveness (EXP(B) = 9.835, *p* = 0.002). Descriptions regarding memorability, effectiveness, and intentions are reported in Table 11.

**TABLE 11:** Measure of memorability, effectiveness, and intention descriptions.

	Memorability	Effectiveness	Intention
Low	37.6%	58.4%	11.6%
Medium	26.8%	23.5%	24.5%
High	35.6%	18.1%	63.9%
Goodness of fit	14.781**	14.951**	25.730*
Nagelkerke	0.107	0.112	0.194

**p < 0.005, *p < 0.05.

To establish which condition was more effective in promoting intention toward prevention behaviors, an ordinal logistic regression model was performed. The dependent variables were classified as low, medium, and high (as in the Study 1). We considered the main effects of the experimental conditions, of the risk perception and of the knowledge about prevention behaviors, also taking into account their interactions. We also considered gender as control and its interaction with the conditions.

In relation to intention, 11.6% of the respondents were in the low range, 24.5% were in the medium range, and 63.9% were in the high range. The prediction model demonstrated goodness of fit to our observed data (χ^2^ (26) = 50.229, *p* = 0.003).

As with Study 1, the behavioral intention was associated with the knowledge of prevention behaviors. No main effects of conditions and risk perception were found. Results, reported in Table 12, showed that the exponential growth condition had a lower effect on the participants who had a medium risk perception compared to those who had a high or low-risk perception. Those with a medium knowledge of prevention behaviors are positively influenced by the neutral and the emotional conditions. Gender and its interactions with conditions did not affect the intention.

**TABLE 12:** Ordinal logistic regressions for the behavioral intention (manipulation 2).

	Manipulation 2: behavioral Intention						
		Estimate	SE	OR (95%CI)		EXP(b)	*p*
**Predictors**							
Exp. Cond	Neutral	−2.030	1.631	−5.228	1.167	1.549	0.213
	Emotional	2.320	2.292	−2.172	6.812	1.025	0.311
	Exp. Growth	0.864	1.565	−2.204	3.932	0.305	0.581
	Combined	0(ref)					
Risk Percep	Low	−0.986	1.202	−3.341	1.369	0.673	0.412
	Medium	0.651	1.124	−1.553	2.855	0.335	0.563
	High	0(ref)					
Knowledge	none	−23.182	34570	−67778	67732	0.000	0.999
	min	−3.816	1.517	−6.789	−0.843	6.330	0.012
	med	−1.684	1.009	−3.662	0.294	2.784	0.095
	max	0(ref)					
**Demographics**							
Gender	Male	−1.523	1.328	−4.126	1.081	1.314	0.252
	Female	0(ref)					
**Interactions**							
**Exp. Conditions × Risk Perception**							
	Neutral × Low	−0.172	1.647	−3.399	3.055	0.011	0.917
	Neutral × Medium	0.444	1.670	−2.830	3.717	0.071	0.791
	Neutral × High	0(ref)					
	Emotional × Low	−2.333	2.178	−6.602	1.936	1.148	0.284
	Emotional × Medium	−3.372	2.133	−7.552	0.808	2.499	0.114
	Emotional × High	0(ref)					
	Exp. G. × Low	−1.124	1.583	−4.228	1.979	0.504	0.478
	Exp. G. × Medium	−3.057	1.477	−5.952	−0.161	4.282	0.039
	Exp. G. × High	0(ref)					
	Comb × Low	0(ref)					
	Comb × Medium	0(ref)					
	Comb × High	0(ref)					
**Exp. Conditions × Knowledge**							
	Neutral × None	43.518	45159	−88467	88554	0.000	0.999
	Neutral × Min	24.559	23256	−45555	45605	0.000	0.999
	Neutral × Med	3.713	1.456	0.859	6.567	6.501	0.011
	Neutral × Max	0(ref)					
	Emotional × None	19.843	34570	−67735	67775	0.000	1.000
	Emotional × Min	4.121	1.847	0.502	7.741	4.980	0.026
	Emotional × Med	1.687	1.353	−0.965	4.339	1.555	0.212
	Emotional × Max	0(ref)					
	Exp. G. × Min	3.124	1.801	−0.406	6.654	3.009	0.083
	Exp. G. × Med	0.075	1.235	−2.346	2.497	0.004	0.951
	Exp. G. × Max	0(ref)					
	Comb × None	0(ref)					
	Comb × Min	0(ref)					
	Comb × Med	0(ref)					
	Comb × Max	0(ref)					
**Exp. Conditions × Gender**							
	Neutral × Male	0.533	1.672	−2.744	3.810	0.102	0.750
	Neutral × Female	0(ref)					
	Emotional × Male	0.366	1.611	−2.791	3.524	0.052	0.820
	Emotional × Female	0(ref)					
	Exp. G. × Male	0.967	1.516	−2.004	3.938	0.407	0.524
	Exp. G. × Female	0(ref)					
	Comb × Male	0(ref)					
	Comb × Female	0(ref)					

Further ordinal logistic regression models were conducted, without modifying the dependent variables and reported in Appendix (see Supplementary Material).

## Discussion

The present study aimed at identifying better ways to promote preventive behavior in Italy in particular situations such as during the critical phase between the lockdown and the post-lockdown period. The results demonstrated that each message transmits the point of view of the source, which effect depends on the confidence in the source that implicitly provides information that goes beyond the literal content, and consequently, influences the message received.

Although the scientific community enjoys generally greater trust than the Government, the national sample attributes mainly to the latter the authority necessary to guide its behavior. The scientific community does not seem to attract the same perception of authority and prescriptive efficacy. Abstractly, the scientific community seems to be perceived as more reliable, but in terms of prescriptive effectiveness, the Government is the most influential source. This discrepancy exists probably because of the contradictory messages coming from the interlocutors, and because of the inflation of the often-conflicting evaluations of virologists and epidemiologists occurring on TV programs. While in the period of total lockdown, the Government was a promoter of obligations and injunctions for sanctions; in the period immediately following, it promoted less stringent obligations; and it continued to have an effect when it used injunctive norms. The results, therefore, demonstrate an increased propensity to adopt preventive behavior when it was the Government to issue injunctive norms. Moreover, with respect to the women, men declared less agreement to adopt preventive behaviors when the scientists promoted the message, however this difference did not affect intention. The descriptive norm did not prove effective in demonstrating the weakness of social imitation in the particular situation of this pandemic.

It is interesting to note that, in our study, the level of trust in institutional sources–measured during the lockdown–differs according to the area of contagion and to risk perception. A study conducted by Sibley et al. (2020) with a representative sample of the New Zealand population demonstrated an increase in confidence in institutions–science, politics, and police–during the lockdown. It would be fruitful to investigate further the possible cultural differences between Italy and New Zealand, to understand whether the results of this study demonstrate the specificity of Italy or a more general tendency. In the sample of students, confidence in the university (and the scientific community) was higher than confidence in the Government. In this case, the messages proposed by the Government, through a descriptive norm, provoked less intention to adopt preventive behaviors. However, in terms of their influence on behaviors, the university’s injunctive and descriptive norms and the Government’s injunctive message did not differ. It is interesting to notice that for the critical group with a lower risk perception (less inclined to adopt prevention behavior) the descriptive norms (communicated by both sources), which implicitly convey the risk perception of peers, were as effective as the Government injunctive norm. Hence, it seems that when a descriptive norm is communicated the effectiveness of social influence is not undermined, because in both cases the reference is the group of peers, in which these critical participants recognize themselves, and this leads them to follow their behaviors. Moreover, since the Government’s messages, both injunctive and descriptive, had an effect in promoting preventive behaviors, it seems that a valid solution in relation to low-risk perception participants is the use of a source with a sanctioning nature.

The findings of the manipulation on the content of the messages highlighted that the neutral condition appeared to be the most memorable, for both samples, and the most effective only for the students’ sample. Although Lunn et al. (2020), also found that the control condition was considered more memorable and more effective by participants we found different results for what concerns the most effective content in promoting preventive behaviors. No condition was more effective than any other. There was, in fact, a high intention to adopt protective behaviors regardless of the aspect on which the communication specifically focused. These differences could be partly due to the different time periods in which the two studies were carried out, in fact, Lunn et al. (2020) conducted their survey immediately before the lockdown, while ours was conducted at the end of the lockdown. Due to the significant amount of information conveyed during the lockdown about preventive behavior, the salience of the specific information about emotional or exponential growth aspects may have been reduced.

An interesting aspect to highlight is the effect found both in the national sample and in the students’ sample to an even greater extent, that the perception of being able to infect others including relatives was greater than the perception of being infected one’s self. This happened despite the fact that the participants believe that they had very low chances of contracting the virus. There appears to be a sort of “bias of contagion” in which the perception of contracting the virus is greatly overestimated, as it is logically more likely to contract the virus than to contract the virus and infect others. Even if the probability is not explicitly mentioned in the question, we suspect that the participants think in accordance with probability when answering this question. Ultimately, they do not consider that to infect others, they must necessarily be infected first. One possibility (especially among younger students) is that they may be thinking about being asymptomatic carriers of the disease. Thus, for the young the risks are more to do with them being transmitters (to the old and vulnerable) than to themselves where, even if they experience symptoms, it is very unlikely to be fatal. This phenomenon is interesting and should be further investigated as it could be used in developing public policies for behavioral change.

In the sample of students, we can hypothesize a link between the message focused on the emotion that worked better, and the overestimated perception of infecting others rather than ourselves. It seems, therefore, that for this sample, the emotional aspect activates to a greater extent the attentional resources on behavioral dispositions, increasing the intention to adopt preventive behaviors, not so much not to contract the virus but rather not to infect loved ones. Additionally, the Government and the Ministry of Health have emphasized individual responsibility, and this may have prompted a possible sense of responsibility and greater guilt in the participants.

In this study, a particular focus was placed on the perception of risk, which in the period considered, was no longer determined only by health risk, but was necessarily a complex measure. In general terms, the results demonstrated that the participants who have a higher perception of risk are more willing to engage in preventive behavior.

Previous knowledge of what to do also seems to be a good predictor of preventive behaviors among students. Those who, as a result of the Ministry’s campaigns, were more aware of the correct behaviors to adopt, were also more willing to adopt them after the lockdown. However, identifying staying at home as a necessary behavior to prevent contagion does not seem to be necessarily accompanied by the adoption of this behavior. The behavior of social isolation, to which Italians had been accustomed for 2 months, no longer seemed to be practical for people, and this did not seem to appear with other preventive behavior.

Our study also confirms the need, already expressed by Bicchieri and Dimant (2019), to develop norm-nudge interventions with respect to some elements. It is essential in this regard to stimulate group identity and citizens’ sense of belonging to achieve more successful outcomes. Additionally, when behavior is perceived as contradictory, especially because of conflicting prescriptions depending on the source that promotes them, it is possible to think of joint actions with other behavioral interventions that may be more stable over time (Grüne-Yanoff and Hertwig, 2016). Collaborative communication, with the government leading the scientists, media, universities, and so on in the delivery of the specific and appropriate message, may improve the efficacy of the message.

Finally, our study offers interesting information about the social norms and sources that would be most effective in managing communication correctly in this crucial post-lockdown phase and, in attempting to consider the complexity of a new and uncertain reality, suggests tools for emergency management.

## Data Availability Statement

The raw data supporting the conclusions of this article will be made available by the authors, without undue reservation.

## Ethics Statement

The studies involving human participants were reviewed and approved by Commissione per la Valutazione della Ricerca (CRIP), Università degli Studi di Milano-Bicocca. The patients/participants provided their written informed consent to participate in this study.

## Author Contributions

RV and LM conceived the original idea, directed the project, supervised the project, and with support from VC and LC designed the study. LC, VC, and FP prepared the questionnaire with Qualtrics. VC carried out the data analysis. VC and LC took the lead in writing the manuscript with support from FP, LM, and RV. All authors provided critical feedback, contributed to the interpretation of the results, discussed the results, and contributed to the final manuscript.

## Conflict of Interest

The authors declare that the research was conducted in the absence of any commercial or financial relationships that could be construed as a potential conflict of interest.

## References

[B1] Ali S. H. Foreman J. Tozan Y. Capasso A. Jones A. M. DiClemente R. J. (2020). Trends and predictors of COVID-19 information sources and their relationship with knowledge and beliefs related to the pandemic: nationwide cross-sectional study. *JMIR Public Health Surveillance* 6:e21071. 10.2196/21071 32936775 PMC7546863

[B2] Alon T. M. Doepke M. Olmstead-Rumsey J. Tertilt M. (2020). *The Impact of COVID-19 on Gender Equality (No. w26947).* Cambridge, MA: National Bureau of Economic Research.

[B3] Bagassi M. Macchi L. (2016). “The interpretative function and the emergence of unconscious analytic thought,” in *Cognitive Unconscious and Human Rationality*, eds Macchi L. Bagassi M. Viale R. (Cambridge, MA: MIT Press), 43–76.

[B4] Barrios J. M. Hochberg Y. (2020). *Risk Perception Through the Lens of Politics in the Time of the Covid-19 Pandemic (No. w27008).* Cambridge, MA: National Bureau of Economic Research.

[B5] Baumeister R. F. Bratslavsky E. Finkenauer C. Vohs K. D. (2001). Bad is stronger than good. *Rev. Gen. Psychol.* 5 323–370. 10.1037/1089-2680.5.4.323

[B6] Betsch C. (2020). How behavioural science data helps mitigate the COVID-19 crisis. *Nat. Hum. Behav.* 4 438–438. 10.1038/s41562-020-0866-1 32221514 PMC7101898

[B7] Bicchieri C. Dimant E. (2019). Nudging with care: the risks and benefits of social information. *Public Choice* 1–22. 10.1007/s11127-019-00684-6

[B8] Bilancini E. Boncinelli L. Capraro V. Celadin T. Di Paolo R. (2020). The effect of norm-based messages on reading and understanding COVID-19 pandemic response governmental rules. *arXiv* preprint. Available online at: https://arxiv.org/abs/2005.03998 (accessed February 25, 2021).

[B9] Briscese G. Lacetera N. Macis M. Tonin M. (2020). *Compliance With Covid-19 Social-Distancing Measures in Italy: the Role of Expectations and Duration (No. w26916).* Cambridge, MA: National Bureau of Economic Research. 10.3386/w26916

[B10] Brzezinski A. Kecht V. Van Dijcke D. Wright A. L. (2020). Belief in science influences physical distancing in response to covid-19 lockdown policies. *Paper Presented at University of Chicago, Becker Friedman Institute for Economics Working Paper, (2020-56)*, (Chicago, IL: University of Chicago).

[B11] Chu L. Fung H. H. Tse D. C. Tsang V. H. Zhang H. Mai C. (2021). Obtaining information from different sources matters during the COVID-19 pandemic. *Gerontologist* 61:gnaa222. 10.1093/geront/gnaa222 33388758 PMC7799117

[B12] Chung A. Rimal R. N. (2016). Social norms: a review. *Rev. Commun. Res.* 4 1–28. 10.12840/issn.2255-4165.2016.04.01.008

[B13] Cialdini R. B. Kallgren C. A. Reno R. R. (1991). A focus theory of normative conduct: a theoretical refinement and reevaluation of the role of norms in human behavior. *Adv. Exp. Soc. Psychol.* 24 201–234. 10.1016/s0065-2601(08)60330-5

[B14] Cialdini R. B. Reno R. R. Kallgren C. A. (1990). A focus theory of normative conduct: recycling the concept of norms to reduce littering in public places. *J. Pers. Soc. Psychol.* 58 1015–1026. 10.1037/0022-3514.58.6.1015

[B15] Cooper J. (2007). *Cognitive Dissonance: Fifty Years of a Classic Theory.* Los Angeles, CA: SAGE Publications. 10.4135/9781446214282

[B16] Couso I. Dubois D. (2014). Statistical reasoning with set-valued information: ontic vs. epistemic views. *Int. J. Approximate Reason.* 55 1502–1518. 10.1016/j.ijar.2013.07.002

[B17] Dickie R. Rasmussen S. Cain R. Williams L. MacKay W. (2018). The effects of perceived social norms on handwashing behaviour in students. *Psychol. Health Med.* 23 154–159. 10.1080/13548506.2017.1338736 28592138

[B18] Festinger L. (1957). *A Theory of Cognitive Dissonance*, Vol. 2. Palo Alto, CA: Stanford university press.

[B19] Frenkel S. Alba D. Zhong R. (2020). *Surge of Virus Misinformation Stumps Facebook and Twitter.* New York, NY: The New York Times.

[B20] Fridman I. Lucas N. Henke D. Zigler C. K. (2020). Association between public knowledge about COVID-19, trust in information sources, and adherence to social distancing: cross-sectional survey. *JMIR Public Health Surveillance* 6:e22060. 10.2196/22060 32930670 PMC7511226

[B21] Frijda N. H. (1986). *The Emotions: Studies in Emotion and Social Interaction.* Cambridge: Cambridge University Press.

[B22] Gigerenzer G. Gaissmaier W. (2011). Heuristic decision making. *Annu. Rev. Psychol.* 62 451–482. 10.1146/annurev-psych-120709-145346 21126183

[B23] Gigerenzer G. Todd P. M. the ABC Research Group (1999). *Simple Heuristics That Make Us Smart.* New York, NY: Oxford University Press.

[B24] Goldstein N. J. Cialdini R. B. Griskevicius V. (2008). A room with a viewpoint: using social norms to motivate environmental conservation in hotels. *J. Consum. Res.* 35 472–482. 10.1086/586910

[B25] Grüne-Yanoff T. Hertwig R. (2016). Nudge versus boost: How coherent are policy and theory? *Minds Mac.* 26 149–183. 10.1007/s11023-015-9367-9

[B26] Jenni K. Loewenstein G. (1997). Explaining the identifiable victim effect. *J. Risk Uncertainty* 14 235–257. 10.1023/A:1007740225484

[B27] Lee S. Feeley T. H. (2016). The identifiable victim effect: a meta-analytic review. *Soc. Influence* 11 199–215. 10.1080/15534510.2016.1216891

[B28] Loewenstein G. F. Weber E. U. Hsee C. K. Welch N. (2001). Risk as feelings. *Psychol. Bull.* 127:267. 10.1037/0033-2909.127.2.267 11316014

[B29] Lunn P. D. Timmons S. Barjaková M. Belton C. A. Julienne H. Lavin C. (2020). *Motivating Social Distancing During the Covid-19 Pandemic: An Online Experiment. Working Paper.* Available online at: https://www.esri.ie/pubs/WP658.pdf (accessed February 25, 2021). 10.1016/j.socscimed.2020.113478 33162198

[B30] Macchi L. Bagassi M. (2019). The argumentative and the interpretative functions of thought: two adaptive characteristics of the human cognitive system. *Teorema Revista Internacional de filosofía* 38 87–96.

[B31] Mohamad E. Tham J. S. Ayub S. H. Hamzah M. R. Hashim H. Azlan A. A. (2020). Relationship between COVID-19 information sources and attitudes in battling the pandemic among the Malaysian public: cross-sectional survey study. *J. Med. Internet Res.* 22:e23922. 10.2196/23922 33151897 PMC7674144

[B32] Moreira M. T. Smith L. A. Foxcroft D. (2009). Social norms interventions to reduce alcohol misuse in university or college students. *Cochrane Database Syst. Rev.* CD006748. 10.1002/14651858.CD006748.pub2 19588402

[B33] Motta Zanin G. Gentile E. Parisi A. Spasiano D. (2020). A preliminary evaluation of the public risk perception related to the COVID-19 health emergency in Italy. *Int. J. Environ. Res. Public Health* 17:3024. 10.3390/ijerph17093024 32349253 PMC7246845

[B34] Newell B. R. Rakow T. Yechiam E. Sambur M. (2016). Rare disaster information can increase risk-taking. *Nat. Clim. Change* 6:158. 10.1038/nclimate2822

[B35] Njå O. Solberg Ø Braut G. S. (2017). “Uncertainty—its ontological status and relation to safety,” in *The Illusion of Risk Control*, eds Motet G. Bieder C. (Cham: Springer), 5–21. 10.1007/978-3-319-32939-0_2

[B36] Olivera-La Rosa A. Chuquichambi E. G. Ingram G. P. (2020). Keep your (social) distance: pathogen concerns and social perception in the time of COVID-19. *Pers. Individ. Differ.* 66:110200. 10.1016/j.paid.2020.110200 32834278 PMC7296322

[B37] Peters E. (2017). “Overcoming innumeracy and the use of heuristics when communicating science,” in *The Oxford Handbook of the Science of Science Communication*, eds Jamieson K. H. Kahan D. Scheufele D. A. (Oxford: Oxford University Press), 389.

[B38] Rottenstreich Y. Hsee C. K. (2001). Money, kisses, and electric shocks: on the affective psychology of risk. *Psychol. Sci.* 12 185–190. 10.1111/1467-9280.00334 11437299

[B39] Savage L. J. (1954). *The Foundations of Statistics*, 2nd Edn. New York, NY: Dover.

[B40] Sibley C. G. Greaves L. M. Satherley N. Wilson M. S. Overall N. C. Lee C. H. (2020). Effects of the COVID-19 pandemic and nationwide lockdown on trust, attitudes toward government, and well-being. *Am. Psychol.* 75 618–630. 10.1037/amp0000662 32496074

[B41] Slovic P. Finucane M. L. Peters E. MacGregor D. G. (2004). Risk as analysis and risk as feelings: some thoughts about affect, reason, risk, and rationality. *Risk Analysis Int. J.* 24 311–322. 10.1111/j.0272-4332.2004.00433.x 15078302

[B42] Small D. A. Loewenstein G. (2003). Helping a victim or helping the victim: altruism and identifiability. *J. Risk Uncertainty* 26 5–16. 10.1023/A:1022299422219

[B43] Sunstein C. R. (2014). Nudging: a very short guide. *J. Consum. Policy* 37 583–588. 10.1007/s10603-014-9273-1

[B44] Tierney J. Baumeister R. F. (2019). *The Power of Bad: And How to Overcome It.* Bristol: Allen Lane.

[B45] Tversky A. Kahneman D. (1973). Availability: a heuristic for judging frequency and probability. *Cogn. Psychol.* 5 207–232. 10.1016/0010-0285(73)90033-9

[B46] Van Bavel J. J. Baicker K. Boggio P. S. Capraro V. Cichocka A. Cikara M. (2020). Using social and behavioural science to support COVID-19 pandemic response. *Nat. Hum. Behav.* 4 460–471. 10.1038/s41562-020-0884-z 32355299

[B47] Veraart J. A. Klostermann J. E. M. van Slobbe E. J. J. Kabat P. (2018). Scientific knowledge use and addressing uncertainties about climate change and ecosystem functioning in the Rhine-Meuse-Scheldt estuaries. *Environ. Sci. Policy* 90 148–160. 10.1016/j.envsci.2018.09.009

[B48] Viale R. (2020b). The epistemic uncertainty of Covid-19: failures and successes of heuristics in clinical decision making. *Mind and Society* 1–6. Available online at: https://link.springer.com/article/10.1007%2Fs11299-020-00262-0 (accessed February 25, 2021).

[B49] Viale R. (2020a). *Handbook on Bounded Rationality.* London: Routledge. 10.4324/9781315658353

[B50] Wagenaar W. A. Sagaria S. D. (1975). Misperception of exponential growth. *Attention Percept. Psychophys.* 18 416–422. 10.3758/BF03204114

[B51] Wise T. Zbozinek T. D. Michelini G. Hagan C. C. Mobbs D. (2020). Changes in risk perception and protective behavior during the first week of the COVID-19 pandemic in the United States. *Preprint* 10.31234/osf.io/dz428 PMC7540790 33047037

[B52] Witte K. (1992). Putting the fear back into fear appeals: the extended parallel process model. *Commun. Monogr.* 59 329–349. 10.1080/03637759209376276

