# Supplementary material for: Behavioral Changes After the COVID-19 Lockdown in Italy
Source: Front Psychol. 2021 Mar 10;12:617315. doi: 10.3389/fpsyg.2021.617315 (PMC7987650; doi:10.3389/fpsyg.2021.617315)
Supplement: Supplementary file 1 [file Data_Sheet_1.pdf]

APPENDIX 1

The original questionnaire provided to the students' sample can be viewed at the following link: https://psicologiaunimib.qualtrics.com/jfe/form/SV_9S9BZDFE2cmijnT. However, the link of the questionnaire that was given to the national sample, is not available since an external market research agency, Doxa, carried out the data collection.

APPENDIX 2

For the three risk perception scales: economic/academic risk perception, privacy risk perception, and mobility risk perception, the participants had to answer a number of items (presented in a random order) indicating their degree of agreement on a Likert scale from 1 (completely disagree) to 7 (completely agree), which were the following:

Health Risk scale:

Both samples:

- I think I have it or have had it
- I think I will get it
- I think I might be asymptomatic
- I think I could infect others
- I think I could infect my relatives and parents
- I think I may have come into contact with people who may be infected
- I think I am immune
- I think I could only contract it in a mild form

Economic/academic Risk scales

National sample:

- It is important that the economy restarts.
- In this situation, I would prefer to stay at home rather than go back to work.
- I would rather become poor than get sick.
- Safety distances can also be maintained at work.
- Sectors such as industry cannot stop.
- I would rather lose my job than contract the virus.
- It is right to keep the population safe even at the risk of financial debt.
- Suffering from hunger is worse than contracting the virus.

Students sample:

- It is important that the university reopens.
- In this situation, I prefer to stay at home rather than go back to university.
- Safety distances can also be maintained at the university.
- I prefer to have exams back rather than contract the virus.
- It is right to keep students safe even at the risk of lowering the quality of education.
- Going off course is worse than getting infected.

Mobility Risk scale

Both samples:

Let us talk about ways and means of getting around. What is the probability, in your opinion, of becoming infected:

- By car
- By bus
- By metro
- By train
- By tram
- On foot
- By bike
- With car sharing

Privacy Risk scale

Both samples:

We would like to know your opinion on the recent news about the release of an app for contagion control. The app was chosen by the government to track the movement of people in "phase 2" of the COVID-19 emergency through a system of anonymous codes. It can be downloaded voluntarily, and it is free of charge on your mobile phone.

- It is important that contagions are monitored.
- Nothing is more important than my privacy.
- I feel safe to share my data with this app.
- I am afraid of losing control of my personal data.
- I prefer to be able to move safely, even at the cost of sacrificing my privacy.
- You can solve the situation without using this type of app.
- This type of technology should only be used if it guarantees the confidentiality of our data.
- It is right to keep the population safe even at the risk of being under control.

A general Risk Perception score was created from the following variables (appropriately oriented): the negative representation of COVID-19 infection, non-essential behavior during the lockdown, and after the lockdown, return to ordinary life assessment, health risk perception, economic/academic risk perception, privacy risk perception, and mobility risk perception. The health risk perception was generated from the items 1) I think I will take it, 2) I think I could infect others, 3) I think I could infect relatives and parents, and 4) I think I could contract it only in a mild form.

APPENDIX 3

Further ordinal logistic regression models were conducted, without modifying the dependent variables, and are reported below.

Study 1

Manipulation 1

Agreement with the behavioral norm to minimize verbal exchanges in indoor public places:

On a Likert scale, ranging from 1 to 7, participants responded 1: 4.3%, 2: 3.8%, 3: 6.3%, 4: 18%, 5: 15.8%, 6: 17.3% and 7: 34.8%.

Goodness of fit: $\chi 2$ (37) = 86.830, $p < .001$.

See Table 1 in "supplementary materials": Ordinal logistic regressions for agreement with the behavioral norm in the national sample (manipulation 1)

Intention to minimize verbal exchanges in indoor public places:

On a Likert scale, ranging from 1 to 7, participants responded 1: 5.8%, 2: 3.3%, 3: 4.8%, 4: 9.8%, 5: 16.5%, 6: 22% and 7: 38%.

Goodness of fit: $\chi 2$ (37) = 92.853, $p < .001$.

See Table 2 in "supplementary materials": Ordinal logistic regressions for the behavioral intention in the national sample (manipulation 1)

Manipulation 2

Intention to adopt preventive behaviors:

On a Likert scale, ranging from 1 to 7, participants responded 1: 0.8%, 2: 0.8%, 3: 3.3%, 4: 4.8%, 5: 18.5%, 6: 30.8% and 7: 41.3%.

Goodness of fit: $\chi 2$ (47) = 102.837, $p < .001$.

See Table 3 in "supplementary materials": Ordinal logistic regressions for the behavioral intention in the national sample (manipulation 2)

Study 2

Manipulation 1

Intention to limit leaving home to the minimum necessary in phase 2:

On a Likert scale, ranging from 1 to 7, participants responded 1: 2%, 2: 2%, 3: 5.3%, 4: 2.6%, 5: 15.8%, 6: 30.9% and 7: 41.4%.

Goodness of fit: $\chi2$ (17) = 54.874, $p < .001$.

See Table 4 in "supplementary materials": Ordinal logistic regressions for the behavioral intention in the students' sample (manipulation 1)

Manipulation 2

Intention to adopt preventive behaviors:

On a Likert scale, ranging from 1 to 7, participants responded 1: 0%, 2: 0%, 3: 2%, 4: 4.8%, 5: 21.8%, 6: 43.5% and 7: 27.9%.

Goodness of fit: $\chi2$ (26) = 52.791, $p = .001$.

See Table 5 in "supplementary materials": Ordinal logistic regressions for the behavioral intention in the students' sample (manipulation 2)

Data concerning the national sample are available through the following link: https://drive.google.com/file/d/1iW1UVmR9ngLp1VDDMj-3Rc8A9216gmfS/view.

Data concerning the students' sample are available through the following link: https://drive.google.com/file/d/1LmwdklyeqDYR17wR5TmQCMihr3AJLimR/view
