# Supplementary material for: Behavioral Changes After the COVID-19 Lockdown in Italy
Source: Front Psychol. 2021 Mar 10;12:617315. doi: 10.3389/fpsyg.2021.617315 (PMC7987650; doi:10.3389/fpsyg.2021.617315)
Supplement: Supplementary file 2 [file Table_1.docx]

|  |  | Manipulation 1: Agreement with the Behavioral Norm | | | | | |
| --- | --- | --- | --- | --- | --- | --- | --- |
|  |  | Estimate | SE | OR (95%CI) | | EXP(b) | p |
| Predictors |  |  |  |  | |  |  |
| **Exp. Cond** | Inj_Gov | 1.942 | 1.046 | -0.107 | 3.991 | 3.449 | 0.063 |
|  | Inj_Scient | 0.212 | 0.921 | -1.593 | 2.017 | 0.053 | 0.818 |
|  | Descr | 0.383 | 0.878 | -1.339 | 2.105 | 0.190 | 0.663 |
|  | Impl | 0(ref) | . | . | . | . | . |
| **Risk Percep** | Low | -1.708 | 0.586 | -2.856 | -0.560 | 8.508 | 0.004 |
|  | Medium | -1.450 | 0.500 | -2.430 | -0.471 | 8.421 | 0.004 |
|  | High | 0(ref) | . | . | . | . | . |
| Covariates |  |  |  |  |  |  |  |
| **Trust_Scient** |  | 0.103 | 0.073 | -0.040 | 0.246 | 1.994 | 0.158 |
| **Trust_Gov** |  | 0.179 | 0.065 | 0.051 | 0.307 | 7.558 | 0.006 |
| Demographics |  |  |  |  |  |  |  |
| **Gender** | Male | 1.092 | 0.406 | 0.296 | 1.887 | 7.239 | 0.007 |
|  | Female | 0(ref) | . | . | . | . | . |
| **Age range** | 18-30 | 0.193 | 0.637 | -1.056 | 1.442 | 0.092 | 0.762 |
|  | 31-45 | -0.199 | 0.588 | -1.352 | 0.953 | 0.115 | 0.734 |
|  | 46-60 | -0.309 | 0.565 | -1.417 | 0.799 | 0.299 | 0.585 |
|  | 61-70 | 0(ref) | . | . | . | . | . |
| **Contagion area** | Low | 0.632 | 0.515 | -0.378 | 1.642 | 1.505 | 0.220 |
|  | Medium | 0.480 | 0.465 | -0.432 | 1.392 | 1.063 | 0.302 |
|  | High | 0(ref) | . | . | . | . | . |
| Interactions |  |  |  |  |  |  |  |
| **Exp. Conditions * Risk Perception** | | |  |  |  |  |  |
|  | Inj_Gov * Low | -0.175 | 0.840 | -1.822 | 1.472 | 0.043 | 0.835 |
|  | Inj_Gov * Medium | 0.071 | 0.741 | -1.382 | 1.524 | 0.009 | 0.924 |
|  | Inj_Gov * High | 0(ref) | . | . | . | . | . |
|  | Inj_Scient * Low | 0.658 | 0.785 | -0.880 | 2.197 | 0.703 | 0.402 |
|  | Inj_Scient * Medium | 1.389 | 0.681 | 0.055 | 2.723 | 4.164 | 0.041 |
|  | Inj_Scient * High | 0(ref) | . | . | . | . | . |
|  | Descr * Low | 0.811 | 0.816 | -0.789 | 2.410 | 0.986 | 0.321 |
|  | Descr * Medium | 0.795 | 0.687 | -0.552 | 2.142 | 1.339 | 0.247 |
|  | Descr * High | 0(ref) | . | . | . | . | . |
|  | Impl * Low | 0(ref) | . | . | . | . | . |
|  | Impl * Medium | 0(ref) | . | . | . | . | . |
|  | Impl * High | 0(ref) | . | . | . | . | . |
| **Exp. Conditions * Gender** | |  |  |  |  |  |  |
|  | Inj_Gov * Male | -1.196 | 0.564 | -2.301 | -0.090 | 4.489 | 0.034 |
|  | Inj_Gov * Female | 0(ref) |  |  |  |  |  |
|  | Inj_Scient * Male | -1.569 | 0.558 | -2.662 | -0.476 | 7.915 | 0.005 |
|  | Inj_Scient * Female | 0(ref) |  |  |  |  |  |
|  | Descr * Male | -1.227 | 0.563 | -2.329 | -0.124 | 4.754 | 0.029 |
|  | Descr * Female | 0(ref) | . | . | . | . | . |
|  | Impl * Male | 0(ref) | . | . | . | . | . |
|  | Impl * Female | 0(ref) | . | . | . | . | . |
| **Exp. Conditions * Age range** | |  |  |  |  |  |  |
|  | Inj_Gov * 18-30 | -1.254 | 0.970 | -3.156 | 0.648 | 1.670 | 0.196 |
|  | Inj_Gov * 31-45 | -0.452 | 0.856 | -2.131 | 1.226 | 0.279 | 0.597 |
|  | Inj_Gov * 46-60 | -0.685 | 0.857 | -2.364 | 0.994 | 0.640 | 0.424 |
|  | Inj_Gov * 61-70 | 0(ref) | . | . | . | . | . |
|  | Inj_Scient * 18-30 | -0.509 | 0.961 | -2.393 | 1.376 | 0.280 | 0.597 |
|  | Inj_Scient * 31-45 | 0.109 | 0.822 | -1.502 | 1.720 | 0.018 | 0.894 |
|  | Inj_Scient * 46-60 | -0.251 | 0.823 | -1.864 | 1.361 | 0.093 | 0.760 |
|  | Inj_Scient * 61-70 | 0(ref) | . | . | . | . | . |
|  | Descr * 18-30 | -0.666 | 0.975 | -2.576 | 1.245 | 0.466 | 0.495 |
|  | Descr * 31-45 | 0.002 | 0.854 | -1.671 | 1.675 | 0.000 | 0.998 |
|  | Descr * 46-60 | 0.809 | 0.851 | -0.859 | 2.477 | 0.903 | 0.342 |
|  | Descr * 61-70 | 0(ref) | . | . | . | . | . |
|  | Impl * 18-30 | 0(ref) | . | . | . | . | . |
|  | Impl * 31-45 | 0(ref) | . | . | . | . | . |
|  | Impl * 46-60 | 0(ref) | . | . | . | . | . |
|  | Impl * 61-70 | 0(ref) | . | . | . | . | . |
| **Exp. Conditions * Contagion area** | | |  |  |  |  |  |
|  | Inj_Gov * Low | -0.772 | 0.662 | -2.069 | 0.525 | 1.362 | 0.243 |
|  | Inj_Gov * Medium | -0.633 | 0.703 | -2.011 | 0.744 | 0.812 | 0.367 |
|  | Inj_Gov * High | 0(ref) | . | . | . | . | . |
|  | Inj_Scient * Low | 0.315 | 0.703 | -1.064 | 1.693 | 0.200 | 0.654 |
|  | Inj_Scient * Medium | -0.458 | 0.646 | -1.725 | 0.809 | 0.502 | 0.479 |
|  | Inj_Scient * High | 0(ref) | . | . | . | . | . |
|  | Descr * Low | -1.024 | 0.723 | -2.441 | 0.393 | 2.006 | 0.157 |
|  | Descr * Medium | -0.542 | 0.636 | -1.789 | 0.705 | 0.726 | 0.394 |
|  | Descr * High | 0(ref) | . | . | . | . | . |
|  | Impl * Low | 0(ref) | . | . | . | . | . |
|  | Impl * Medium | 0(ref) | . | . | . | . | . |
|  | Impl * High | 0(ref) | . | . | . | . | . |
