# Supplementary material for: Behavioral Changes After the COVID-19 Lockdown in Italy
Source: Front Psychol. 2021 Mar 10;12:617315. doi: 10.3389/fpsyg.2021.617315 (PMC7987650; doi:10.3389/fpsyg.2021.617315)
Supplement: Supplementary file 3 [file Table_2.docx]

|  |  | Manipulation 1: Intention | | | | | |
| --- | --- | --- | --- | --- | --- | --- | --- |
|  |  | Estimate | SE | OR (95%CI) | | EXP(b) | p |
| Predictors |  |  |  |  | |  |  |
| **Exp. Cond** | Inj_Gov | 1.934 | 1.022 | -0.069 | 3.936 | 3.582 | 0.058 |
|  | Inj_Scient | 0.398 | 0.882 | -1.330 | 2.126 | 0.204 | 0.652 |
|  | Descr | 0.502 | 0.880 | -1.224 | 2.227 | 0.325 | 0.569 |
|  | Impl | 0(ref) | . | . | . | . | . |
| **Risk Percep** | Low | -1.040 | 0.519 | -2.056 | -0.024 | 4.023 | 0.045 |
|  | Medium | -0.449 | 0.435 | -1.302 | 0.404 | 1.063 | 0.302 |
|  | High | 0(ref) | . | . | . | . | . |
| Covariates |  |  |  |  |  |  |  |
| **Trust_Scient** |  | 0.180 | 0.074 | 0.035 | 0.325 | 5.913 | 0.015 |
| **Trust_Gov** |  | 0.007 | 0.064 | -0.119 | 0.132 | 0.011 | 0.918 |
| Demographics |  |  |  |  |  |  |  |
| **Gender** | Male | 0.224 | 0.365 | -0.491 | 0.939 | 0.377 | 0.539 |
|  | Female | 0(ref) | . | . | . | . | . |
| **Age range** | 18-30 | -0.816 | 0.604 | -2.000 | 0.369 | 1.822 | 0.177 |
|  | 31-45 | 0.012 | 0.550 | -1.065 | 1.090 | 0.001 | 0.982 |
|  | 46-60 | -0.668 | 0.512 | -1.672 | 0.336 | 1.700 | 0.192 |
|  | 61-70 | 0(ref) | . | . | . | . | . |
| **Contagion area** | Low | -0.646 | 0.461 | -1.549 | 0.257 | 1.965 | 0.161 |
|  | Medium | -0.140 | 0.432 | -0.987 | 0.708 | 0.105 | 0.746 |
|  | High | 0(ref) | . | . | . | . | . |
| Interactions |  |  |  |  |  |  |  |
| **Exp. Conditions * Risk Perception** | | |  |  |  |  |  |
|  | Inj_Gov * Low | -1.381 | 0.834 | -3.015 | 0.253 | 2.743 | 0.098 |
|  | Inj_Gov * Medium | -1.628 | 0.732 | -3.064 | -0.193 | 4.942 | 0.026 |
|  | Inj_Gov * High | 0(ref) | . | . | . | . | . |
|  | Inj_Scient * Low | -0.497 | 0.732 | -1.933 | 0.938 | 0.461 | 0.497 |
|  | Inj_Scient * Medium | -0.013 | 0.633 | -1.255 | 1.228 | 0.000 | 0.983 |
|  | Inj_Scient * High | 0(ref) | . | . | . | . | . |
|  | Descr * Low | -1.001 | 0.794 | -2.558 | 0.555 | 1.590 | 0.207 |
|  | Descr * Medium | -0.334 | 0.651 | -1.609 | 0.942 | 0.263 | 0.608 |
|  | Descr * High | 0(ref) | . | . | . | . | . |
|  | Impl * Low | 0(ref) | . | . | . | . | . |
|  | Impl * Medium | 0(ref) | . | . | . | . | . |
|  | Impl * High | 0(ref) | . | . | . | . | . |
| **Exp. Conditions * Gender** | |  |  |  |  |  |  |
|  | Inj_Gov * Male | -0.417 | 0.535 | -1.465 | 0.631 | 0.608 | 0.435 |
|  | Inj_Gov * Female | 0(ref) | . | . | . | . | . |
|  | Inj_Scient * Male | -0.867 | 0.526 | -1.898 | 0.164 | 2.718 | 0.099 |
|  | Inj_Scient * Female | 0(ref) | . | . | . | . | . |
|  | Descr * Male | -0.543 | 0.546 | -1.613 | 0.527 | 0.990 | 0.320 |
|  | Descr * Female | 0(ref) | . | . | . | . | . |
|  | Impl * Male | 0(ref) | . | . | . | . | . |
|  | Impl * Female | 0(ref) | . | . | . | . | . |
| **Exp. Conditions * Age range** | |  |  |  |  |  |  |
|  | Inj_Gov * 18-30 | -0.270 | 0.935 | -2.103 | 1.563 | 0.083 | 0.773 |
|  | Inj_Gov * 31-45 | -0.204 | 0.805 | -1.782 | 1.374 | 0.064 | 0.800 |
|  | Inj_Gov * 46-60 | -0.428 | 0.800 | -1.996 | 1.139 | 0.287 | 0.592 |
|  | Inj_Gov * 61-70 | 0(ref) | . | . | . | . | . |
|  | Inj_Scient * 18-30 | -0.134 | 0.907 | -1.912 | 1.643 | 0.022 | 0.882 |
|  | Inj_Scient * 31-45 | -0.135 | 0.772 | -1.649 | 1.379 | 0.030 | 0.861 |
|  | Inj_Scient * 46-60 | 0.879 | 0.770 | -0.630 | 2.387 | 1.304 | 0.254 |
|  | Inj_Scient * 61-70 | 0(ref) | . | . | . | . | . |
|  | Descr * 18-30 | 0.026 | 0.957 | -1.850 | 1.901 | 0.001 | 0.979 |
|  | Descr * 31-45 | -0.663 | 0.831 | -2.292 | 0.966 | 0.637 | 0.425 |
|  | Descr * 46-60 | 0.873 | 0.822 | -0.737 | 2.483 | 1.130 | 0.288 |
|  | Descr * 61-70 | 0(ref) | . | . | . | . | . |
|  | Impl * 18-30 | 0(ref) | . | . | . | . | . |
|  | Impl * 31-45 | 0(ref) | . | . | . | . | . |
|  | Impl * 46-60 | 0(ref) | . | . | . | . | . |
|  | Impl * 61-70 | 0(ref) | . | . | . | . | . |
| **Exp. Conditions * Contagion area** | | |  |  |  |  |  |
|  | Inj_Gov * Low | 0.588 | 0.622 | -0.631 | 1.806 | 0.893 | 0.345 |
|  | Inj_Gov * Medium | -0.553 | 0.659 | -1.844 | 0.739 | 0.704 | 0.402 |
|  | Inj_Gov * High | 0(ref) | . | . | . | . | . |
|  | Inj_Scient * Low | 0.763 | 0.644 | -0.499 | 2.026 | 1.404 | 0.236 |
|  | Inj_Scient * Medium | 0.570 | 0.645 | -0.694 | 1.835 | 0.781 | 0.377 |
|  | Inj_Scient * High | 0(ref) | . | . | . | . | . |
|  | Descr * Low | 1.396 | 0.704 | 0.016 | 2.776 | 3.931 | 0.047 |
|  | Descr * Medium | 0.133 | 0.637 | -1.116 | 1.383 | 0.044 | 0.835 |
|  | Descr * High | 0(ref) | . | . | . | . | . |
|  | Impl * Low | 0(ref) | . | . | . | . | . |
|  | Impl * Medium | 0(ref) | . | . | . | . | . |
|  | Impl * High | 0(ref) | . | . | . | . | . |
