# Supplementary material for: Behavioral Changes After the COVID-19 Lockdown in Italy
Source: Front Psychol. 2021 Mar 10;12:617315. doi: 10.3389/fpsyg.2021.617315 (PMC7987650; doi:10.3389/fpsyg.2021.617315)
Supplement: Supplementary file 4 [file Table_3.docx]

|  |  | Manipulation 2: behavioral Intention | | | | | |
| --- | --- | --- | --- | --- | --- | --- | --- |
|  |  | Estimate | SE | OR (95%CI) | | EXP(b) | p |
| Predictors |  |  |  |  | |  |  |
| **Exp. Cond** | Neutral | 0.985 | 1.174 | -1.316 | 3.285 | 0.704 | 0.402 |
|  | Emotional | -1.392 | 1.039 | -3.428 | 0.644 | 1.797 | 0.180 |
|  | Exp. Growth | -0.639 | 1.059 | -2.716 | 1.437 | 0.364 | 0.546 |
|  | Combined | 0(ref) | . | . | . | . | . |
| **Risk Percep** | Low | -1.135 | 0.598 | -2.308 | 0.037 | 3.603 | 0.058 |
|  | Medium | -1.121 | 0.523 | -2.145 | -0.096 | 4.594 | 0.032 |
|  | High | 0(ref) | . | . | . | . | . |
| **Knowledge** | none | -0.850 | 1.158 | -3.120 | 1.421 | 0.538 | 0.463 |
|  | min | -0.729 | 0.594 | -1.893 | 0.434 | 1.508 | 0.219 |
|  | med | -0.353 | 0.435 | -1.205 | 0.500 | 0.658 | 0.417 |
|  | max | 0(ref) | . | . | . | . | . |
| Demographics |  |  |  |  |  |  |  |
| **Gender** | Male | -0.453 | 0.413 | -1.263 | 0.356 | 1.204 | 0.272 |
|  | Female | 0(ref) | . | . | . | . | . |
| **Age range** | 18-30 | -0.574 | 0.708 | -1.962 | 0.813 | 0.658 | 0.417 |
|  | 31-45 | -0.170 | 0.635 | -1.415 | 1.075 | 0.072 | 0.789 |
|  | 46-60 | -0.343 | 0.617 | -1.551 | 0.865 | 0.310 | 0.578 |
|  | 61-70 | 0(ref) | . | . | . | . | . |
| **Contagion area** | Low | -0.605 | 0.500 | -1.586 | 0.376 | 1.460 | 0.227 |
|  | Medium | -0.071 | 0.487 | -1.025 | 0.883 | 0.021 | 0.884 |
|  | High | 0(ref) | . | . | . | . | . |
| Interactions |  |  |  |  |  |  |  |
| **Exp. Conditions * Risk Perception** | | |  |  |  |  |  |
|  | Neutral * Low | -0.843 | 0.919 | -2.645 | 0.959 | 0.841 | 0.359 |
|  | Neutral * Medium | -0.172 | 0.821 | -1.782 | 1.438 | 0.044 | 0.835 |
|  | Neutral * High | 0(ref) | . | . | . | . | . |
|  | Emotional * Low | -0.135 | 0.809 | -1.721 | 1.451 | 0.028 | 0.867 |
|  | Emotional * Medium | 0.696 | 0.700 | -0.675 | 2.068 | 0.990 | 0.320 |
|  | Emotional * High | 0(ref) | . | . | . | . | . |
|  | Exp. G. * Low | 0.562 | 0.859 | -1.121 | 2.245 | 0.428 | 0.513 |
|  | Exp. G. * Medium | 1.406 | 0.710 | 0.016 | 2.797 | 3.928 | 0.047 |
|  | Exp. G.* High | 0(ref) | . | . | . | . | . |
|  | Comb * Low | 0(ref) | . | . | . | . | . |
|  | Comb * Medium | 0(ref) | . | . | . | . | . |
|  | Comb * High | 0(ref) | . | . | . | . | . |
| **Exp. Conditions * Knowledge** | |  |  |  |  |  |  |
|  | Neutral * None | -0.821 | 1.567 | -3.892 | 2.251 | 0.274 | 0.601 |
|  | Neutral * Min | -0.755 | 0.865 | -2.450 | 0.939 | 0.763 | 0.382 |
|  | Neutral * Med | -0.151 | 0.659 | -1.443 | 1.142 | 0.052 | 0.819 |
|  | Neutral * Max | 0(ref) | . | . | . | . | . |
|  | Emotional * None | -2.392 | 1.590 | -5.509 | 0.725 | 2.263 | 0.133 |
|  | Emotional * Min | -0.827 | 0.827 | -2.448 | 0.793 | 1.001 | 0.317 |
|  | Emotional * Med | 0.286 | 0.622 | -0.933 | 1.505 | 0.212 | 0.645 |
|  | Emotional * Max | 0(ref) | . | . | . | . | . |
|  | Exp. G. * None | -0.433 | 1.426 | -3.227 | 2.362 | 0.092 | 0.762 |
|  | Exp. G. * Min | -0.060 | 0.864 | -1.753 | 1.633 | 0.005 | 0.945 |
|  | Exp. G.* Med | 0.624 | 0.635 | -0.621 | 1.869 | 0.964 | 0.326 |
|  | Exp. G.* Max | 0(ref) | . | . | . | . | . |
|  | Comb * None | 0(ref) | . |  |  |  |  |
|  | Comb * Min | 0(ref) | . | . | . | . | . |
|  | Comb * Med | 0(ref) | . | . | . | . | . |
|  | Comb * Max | 0(ref) | . | . | . | . | . |
| **Exp. Conditions * Gender** | |  |  |  |  |  |  |
|  | Neutral * Male | -0.151 | 0.596 | -1.319 | 1.017 | 0.064 | 0.800 |
|  | Neutral * Female | 0(ref) | . | . | . | . | . |
|  | Emotional * Male | -0.156 | 0.574 | -1.281 | 0.969 | 0.074 | 0.786 |
|  | Emotional * Female | 0(ref) | . | . | . | . | . |
|  | Exp. G. * Male | -0.033 | 0.574 | -1.157 | 1.091 | 0.003 | 0.954 |
|  | Exp. G. * Female | 0(ref) | . | . | . | . | . |
|  | Comb * Male | 0(ref) | . | . | . | . | . |
|  | Comb * Female | 0(ref) | . | . | . | . | . |
| **Exp. Conditions * Age range** | |  |  |  |  |  |  |
|  | Neutral * 18-30 | -1.118 | 1.015 | -3.107 | 0.870 | 1.215 | 0.270 |
|  | Neutral * 31-45 | -0.981 | 0.874 | -2.694 | 0.732 | 1.260 | 0.262 |
|  | Neutral * 46-60 | -0.156 | 0.892 | -1.904 | 1.592 | 0.031 | 0.861 |
|  | Neutral * 61-70 | 0(ref) | . | . | . | . | . |
|  | Emotional * 18-30 | 0.074 | 0.994 | -1.873 | 2.022 | 0.006 | 0.940 |
|  | Emotional * 31-45 | 0.303 | 0.864 | -1.390 | 1.996 | 0.123 | 0.726 |
|  | Emotional * 46-60 | 0.599 | 0.854 | -1.074 | 2.272 | 0.492 | 0.483 |
|  | Emotional * 61-70 | 0(ref) | . | . | . | . | . |
|  | Exp. G. * 18-30 | -1.105 | 1.039 | -3.141 | 0.931 | 1.132 | 0.287 |
|  | Exp. G. * 31-45 | -1.438 | 0.922 | -3.246 | 0.369 | 2.433 | 0.119 |
|  | Exp. G. * 46-60 | -0.106 | 0.930 | -1.928 | 1.715 | 0.013 | 0.909 |
|  | Exp. G. * 61-70 | 0(ref) | . | . | . | . | . |
|  | Comb * 18-30 | 0(ref) | . | . | . | . | . |
|  | Comb * 31-45 | 0(ref) | . | . | . | . | . |
|  | Comb * 46-60 | 0(ref) | . | . | . | . | . |
|  | Comb * 61-70 | 0(ref) | . | . | . | . | . |
| **Exp. Conditions * Contagion area** | | |  |  |  |  |  |
|  | Neutral * Low | 0.387 | 0.674 | -0.934 | 1.709 | 0.330 | 0.565 |
|  | Neutral * Medium | 0.004 | 0.734 | -1.435 | 1.443 | 0.000 | 0.996 |
|  | Neutral * High | 0(ref) | . | . | . | . | . |
|  | Emotional * Low | 0.318 | 0.708 | -1.070 | 1.705 | 0.201 | 0.654 |
|  | Emotional * Medium | 0.973 | 0.684 | -0.367 | 2.313 | 2.024 | 0.155 |
|  | Emotional * High | 0(ref) | . | . | . | . | . |
|  | Exp. G. * Low | 0.994 | 0.741 | -0.460 | 2.447 | 1.796 | 0.180 |
|  | Exp. G. * Medium | -0.180 | 0.678 | -1.508 | 1.149 | 0.070 | 0.791 |
|  | Exp. G.* High | 0(ref) | . | . | . | . | . |
|  | Comb * Low | 0(ref) | . | . | . | . | . |
|  | Comb * Medium | 0(ref) | . | . | . | . | . |
|  | Comb * High | 0(ref) | . | . | . | . | . |
