# Supplementary material for: Behavioral Changes After the COVID-19 Lockdown in Italy
Source: Front Psychol. 2021 Mar 10;12:617315. doi: 10.3389/fpsyg.2021.617315 (PMC7987650; doi:10.3389/fpsyg.2021.617315)
Supplement: Supplementary file 5 [file Table_4.docx]

|  |  | Manipulation 1: Intention | | | | | |
| --- | --- | --- | --- | --- | --- | --- | --- |
|  |  | Estimate | SE | OR (95%CI) | | EXP(b) | p |
| Predictors |  |  |  |  | |  |  |
| **Exp. Cond** | Descr_Gov | -2.845 | 1.277 | -5.348 | -0.343 | 4.965 | 0.026 |
|  | Descr_Univ | -1.493 | 1.312 | -4.064 | 1.077 | 1.296 | 0.255 |
|  | Inj_Gov | -1.274 | 1.325 | -3.871 | 1.322 | 0.925 | 0.336 |
|  | Inj_Univ | 0(ref) | . | . | . | . | . |
| **Risk Percep** | Low | -5.631 | 1.261 | -8.104 | -3.159 | 19.932 | 0.000 |
|  | Medium | -2.572 | 1.196 | -4.915 | -0.228 | 4.627 | 0.031 |
|  | High | 0(ref) | . | . | . | . | . |
| Covariates |  |  |  |  |  |  |  |
| **Trust_Univ** |  | 0.120 | 0.154 | -0.182 | 0.422 | 0.605 | 0.437 |
| **Trust_Gov** |  | 0.283 | 0.114 | 0.060 | 0.506 | 6.164 | 0.013 |
| Demographics |  |  |  |  |  |  |  |
| **Gender** | Male | 0.618 | 0.823 | -0.996 | 2.232 | 0.563 | 0.453 |
|  | Female | 0(ref) | . | . | . | . | . |
| Interactions |  |  |  |  |  |  |  |
| **Exp. Conditions * Risk Perception** | | |  |  |  |  |  |
|  | Descr_Gov * Low | 4.205 | 1.517 | 1.232 | 7.178 | 7.683 | 0.006 |
|  | Descr_Gov * Medium | 1.862 | 1.426 | -0.933 | 4.656 | 1.705 | 0.192 |
|  | Descr_Gov * High | 0(ref) | . | . | . | . | . |
|  | Descr_Univ * Low | 3.413 | 1.555 | 0.366 | 6.460 | 4.821 | 0.028 |
|  | Descr_Univ * Medium | 1.760 | 1.463 | -1.108 | 4.628 | 1.446 | 0.229 |
|  | Descr_Univ * High | 0(ref) | . | . | . | . | . |
|  | Inj_Gov * Low | 3.518 | 1.586 | 0.410 | 6.626 | 4.923 | 0.026 |
|  | Inj_Gov * Medium | 1.438 | 1.419 | -1.343 | 4.219 | 1.027 | 0.311 |
|  | Inj_Gov * High | 0(ref) | . | . | . | . | . |
|  | Inj_Univ * Low | 0(ref) | . | . | . | . | . |
|  | Inj_Univ * Medium | 0(ref) | . | . | . | . | . |
|  | Inj_Univ * High | 0(ref) | . | . | . | . | . |
| **Exp. Conditions * Gender** | |  |  |  |  |  |  |
|  | Descr_Gov * Male | -1.184 | 1.315 | -3.761 | 1.393 | 0.811 | 0.368 |
|  | Descr_Gov * Female | 0(ref) | . | . | . | . | . |
|  | Descr_Univ* Male | -1.435 | 1.114 | -3.618 | 0.749 | 1.658 | 0.198 |
|  | Descr_Univ * Female | 0(ref) | . | . | . | . | . |
|  | Inj_Gov * Male | -1.356 | 1.047 | -3.407 | 0.696 | 1.676 | 0.195 |
|  | Inj_Gov * Female | 0(ref) | . | . | . | . | . |
|  | Inj_Univ * Male | 0(ref) | . | . | . | . | . |
|  | Inj_Univ* Female | 0(ref) | . | . | . | . | . |
