# Supplementary material for: Behavioral Changes After the COVID-19 Lockdown in Italy
Source: Front Psychol. 2021 Mar 10;12:617315. doi: 10.3389/fpsyg.2021.617315 (PMC7987650; doi:10.3389/fpsyg.2021.617315)
Supplement: Supplementary file 6 [file Table_5.docx]

|  |  | Manipulation 2: behavioral Intention | | | | | |
| --- | --- | --- | --- | --- | --- | --- | --- |
|  |  | Estimate | SE | OR (95%CI) | | EXP(b) | p |
| Predictors |  |  |  |  | |  |  |
| **Exp. Cond** | Neutral | 0.653 | 1.353 | -1.999 | 3.305 | 0.233 | 0.630 |
|  | Emotional | 1.083 | 1.385 | -1.632 | 3.797 | 0.611 | 0.434 |
|  | Exp. Growth | 0.866 | 1.179 | -1.445 | 3.177 | 0.539 | 0.463 |
|  | Combined | 0(ref) | . | . | . | . | . |
| **Risk Percep** | Low | -1.503 | 1.055 | -3.571 | 0.564 | 2.030 | 0.154 |
|  | Medium | -0.609 | 0.942 | -2.455 | 1.236 | 0.419 | 0.518 |
|  | High | 0(ref) | . | . | . | . | . |
| **Knowledge** | none | -2.329 | 1.981 | -6.212 | 1.554 | 1.382 | 0.240 |
|  | min | -2.539 | 1.180 | -4.851 | -0.227 | 4.633 | 0.031 |
|  | med | -1.042 | 0.751 | -2.513 | 0.430 | 1.925 | 0.165 |
|  | max | 0(ref) | . | . | . | . | . |
| Demographics |  |  |  |  |  |  |  |
| **Gender** | Male | -0.955 | 1.025 | -2.965 | 1.054 | 0.868 | 0.352 |
|  | Female | 0(ref) | . | . | . | . | . |
| Interactions |  |  |  |  |  |  |  |
| **Exp. Conditions * Risk Perception** | | |  |  |  |  |  |
|  | Neutral * Low | -1.250 | 1.536 | -4.262 | 1.761 | 0.662 | 0.416 |
|  | Neutral * Medium | -0.371 | 1.357 | -3.030 | 2.288 | 0.075 | 0.784 |
|  | Neutral * High | 0(ref) | . | . | . | . | . |
|  | Emotional * Low | -0.904 | 1.455 | -3.755 | 1.946 | 0.387 | 0.534 |
|  | Emotional * Medium | -0.904 | 1.336 | -3.523 | 1.715 | 0.458 | 0.499 |
|  | Emotional * High | 0(ref) | . | . | . | . | . |
|  | Exp. G. * Low | -0.673 | 1.380 | -3.379 | 2.033 | 0.238 | 0.626 |
|  | Exp. G. * Medium | -2.221 | 1.209 | -4.591 | 0.148 | 3.376 | 0.066 |
|  | Exp. G.* High | 0(ref) | . | . | . | . | . |
|  | Comb * Low | 0(ref) | . | . | . | . | . |
|  | Comb * Medium | 0(ref) | . | . | . | . | . |
|  | Comb * High | 0(ref) | . | . | . | . | . |
| **Exp. Conditions * Knowledge** | |  |  |  |  |  |  |
|  | Neutral * None | 2.962 | 2.496 | -1.931 | 7.854 | 1.408 | 0.235 |
|  | Neutral * Min | 2.214 | 1.682 | -1.081 | 5.510 | 1.734 | 0.188 |
|  | Neutral * Med | 1.669 | 1.126 | -0.538 | 3.877 | 2.197 | 0.138 |
|  | Neutral * Max | 0(ref) | . | . | . | . | . |
|  | Emotional * None | -1.057 | 2.284 | -5.534 | 3.420 | 0.214 | 0.644 |
|  | Emotional * Min | 4.222 | 1.562 | 1.161 | 7.282 | 7.309 | 0.007 |
|  | Emotional * Med | 1.286 | 1.017 | -0.707 | 3.278 | 1.599 | 0.206 |
|  | Emotional * Max | 0(ref) | . | . | . | . | . |
|  | Exp. G. * Min | 2.234 | 1.521 | -0.748 | 5.216 | 2.156 | 0.142 |
|  | Exp. G.* Med | 0.063 | 1.006 | -1.908 | 2.034 | 0.004 | 0.950 |
|  | Exp. G.* Max | 0(ref) | . | . | . | . | . |
|  | Comb * None | 0(ref) | . | . | . | . | . |
|  | Comb * Min | 0(ref) | . | . | . | . | . |
|  | Comb * Med | 0(ref) | . | . | . | . | . |
|  | Comb * Max | 0(ref) | . | . | . | . | . |
| **Exp. Conditions * Gender** | |  |  |  |  |  |  |
|  | Neutral * Male | -0.260 | 1.308 | -2.824 | 2.303 | 0.040 | 0.842 |
|  | Neutral * Female | 0(ref) | . | . | . | . | . |
|  | Emotional * Male | -0.498 | 1.315 | -3.075 | 2.079 | 0.143 | 0.705 |
|  | Emotional * Female | 0(ref) | . | . | . | . | . |
|  | Exp. G. * Male | 0.352 | 1.266 | -2.129 | 2.832 | 0.077 | 0.781 |
|  | Exp. G. * Female | 0(ref) | . | . | . | . | . |
|  | Comb * Male | 0(ref) | . | . | . | . | . |
|  | Comb * Female | 0(ref) | . | . | . | . | . |
